# Immunophenotyping monocytes, macrophages and granulocytes in the Pteropodid bat *Eonycteris spelaea*

**DOI:** 10.1038/s41598-019-57212-1

**Published:** 2020-01-15

**Authors:** Akshamal M. Gamage, Feng Zhu, Matae Ahn, Randy Jee Hiang Foo, Ying Ying Hey, Dolyce H. W. Low, Ian H. Mendenhall, Charles-Antoine Dutertre, Lin-Fa Wang

**Affiliations:** 1 Programme in Emerging Infectious Diseases, Duke-NUS Medical School, Singapore, Singapore; 2 Singapore Immunology Network (SIgN), Agency for Science Technology and Research (A*STAR), Singapore, Singapore

**Keywords:** Viral infection, Viral infection, Innate immunity, Innate immunity

## Abstract

Bats are asymptomatic reservoir hosts for several highly pathogenic viruses. Understanding this enigmatic relationship between bats and emerging zoonotic viruses requires tools and approaches which enable the comparative study of bat immune cell populations and their functions. We show that bat genomes have a conservation of immune marker genes which delineate phagocyte populations in humans, while lacking key mouse surface markers such as Ly6C and Ly6G. Cross-reactive antibodies against CD44, CD11b, CD14, MHC II, and CD206 were multiplexed to characterize circulating monocytes, granulocytes, bone-marrow derived macrophages (BMDMs) and lung alveolar macrophages (AMs) in the cave nectar bat *Eonycteris spelaea*. Transcriptional profiling of bat monocytes and BMDMs identified additional markers – including MARCO, CD68, CD163, CD172α, and CD88 – which can be used to further characterize bat myeloid populations. Bat cells often resembled their human counterparts when comparing immune parameters that are divergent between humans and mice, such as the expression patterns of certain immune cell markers. A genome-wide comparison of immune-related genes also revealed a much closer phylogenetic relationship between bats and humans compared to rodents. Taken together, this study provides a set of tools and a comparative framework which will be important for unravelling viral disease tolerance mechanisms in bats.

## Introduction

Bats, belonging to the order Chiroptera, are the only mammals capable of powered flight. In the mammalian tree, Chiropterans are placed within the super-order Laurasiatheria and their close extant relatives include the carnivores and ungulates^1^. Phylogenetic analysis has further classified bats into two suborders – the Yinpterochiroptera which encompass the non-echolocating Old-world fruit bats and one microbat lineage (Rhinolophidae), and the Yangochiroptera which includes the rest of the echolocating microbats^1,2^. The cave nectar bat *Eonycteris spelaea* utilized in this study is a pteropodid bat within the Yinpterochiroptera lineage, and has a broad geographical distribution across South and South East Asia^3^. Bats are key ecosystem service providers, acting as pollinators, dispersing fruits and seeds, and controlling insects of agricultural and public health importance^4^. Bat species from both suborders are also reservoir hosts to a wide range of highly lethal zoonotic viruses with pathogenic potential in both humans and livestock^5,6^. Hence there is a significant interest in understanding the unique host biology responsible for the ability of bats to harbor pathogenic viruses asymptomatically^7^.

A prevailing theory for the co-existence of bats and viruses is that adapting to physiological stressors associated with the evolution of powered flight necessitated a re-balancing of the chiropteran immune system, with consequent effects on viral disease tolerance and infection-associated immunopathology. In line with this hypothesis, several intracellular sensors capable of detecting endogenously derived danger signals have a reduced functionality or are absent in bats, with evidence from species in both bat suborders^7–9^. Further mechanistic studies are required to determine if there is indeed a unifying explanation linking the evolution of flight to viral disease tolerance, and perhaps to the exceptional longevity seen in bats as well. Studying bat immunology is also important for understanding the response of the natural reservoir to bat-borne zoonotic viruses, and can offer fresh perspectives in modulating the immune response during infection as a therapeutic strategy in patients. Thus there is an important need to establish *in-vitro* tools to further facilitate the study of bat immunology.

Monocytes, macrophages and circulating granulocytes are the professional phagocytes in the vertebrate immune system^10^. This group of cells is of particular interest in bat immunology as they are responsible for several effector functions – in addition to phagocytosis – which are intimately linked to viral control and immunopathology. These functions include interferon signaling, antigen presentation, inflammasome activation, and the production of reactive oxygen species (ROS) and other inflammatory mediators^11^. They are also key innate immune cells involved in the early host response to infection and could be expected to play an important role in shaping the nature and extent of the overall immune response in bats during infection.

Circulating granulocytes are primarily composed of polymorphonuclear neutrophils (PMNs). These are short-lived cells which are constantly replenished from the bone marrow by granulopoiesis^12^. Upon pathogen encounter, PMNs degranulate to release a cocktail of microbicidal effectors, and produce a powerful respiratory burst to inactivate phagocytosed pathogens^13^. PMN degranulation and ROS production can also cause significant collateral damage to host tissue. Monocytes and macrophages, together with dendritic cells (DCs), comprise the mononuclear phagocyte system (MPS)^14^. Cells of the MPS are longer lived, and exhibit significant heterogeneity and plasticity, both functionally and phenotypically^15^. Cells of the MPS play an extensive role in protecting the host from infection due to their innate immune sentinel capacity, ability to co-ordinate the development of an adaptive immune response, and involvement in tissue repair and remodeling^16^. However, they too can contribute to immunopathology and the adverse resolution of inflammation – such as driving organ fibrosis^17^ – if their functions are not restrained.

In this study, we demonstrate immunophenotyping approaches to characterize various phagocyte populations in bats. The methods described herein enable the further study of isolated bat myeloid cells *in vitro* and can also be utilized as part of a broader interrogation of the *in vivo* immune response in bats during experimental infection.

## Results

### Conservation of myeloid cell markers between humans and bats

To identify potential surface markers for experimental validation, we first evaluated bat genomes (two each from Yinpterochiroptera and Yangochiroptera) for homologs of human and mouse myeloid markers. Mouse circulating monocytes are commonly identified on the basis of Ly6C, Ly6G, CCR2 and CX3CR1 expression – “inflammatory” monocytes are described as Ly6C^+^ Ly6G^−^ CCR2^+^ CX3CR1^l^°^w^, while “resident” monocytes are Ly6C^l^°^w^ Ly6G^−^ CCR2^−^ CX3CR1^+ ^^18^. The Ly6 antigen is also useful for identifying circulating mouse neutrophils (Ly6C^+^ Ly6G^+^)^19^. The chemokine receptors CCR2 and CX3CR1 have equivalent gene homologs in bats (Table 1). However, Ly6C and Ly6G belong to the lymphocyte antigen-6/urokinase-type plasminogen activator (uPar) superfamily of proteins, which have multiple paralogs within species but often lack clear one-to-one orthologs across species^20^. The bat genomes examined did not possess orthologs of Ly6C and Ly6G (Table 1).

**Table 1:** Amino acid identity of myeloid marker homologs in humans, mice and bats.

Surface marker	% amino acid identity					
	*H. sapiens*	*M. musculus*	*P. alecto*	*E. spelaea*	*M. davidii*	*E. fuscus*
CD11b	100	75	78	78	78	74
CD14	100	66	68	66	66	70
CD16(A)	100	61	61	63	54	54
CD206	100	82	87	86	85	86
CD172α	100	65	75	65	71	74
Ly6C	x	100	x	x	x	x
Ly6G	x	100	x	x	x	x
F4/80	60	100	63	68	68	68
CCR2	100	77	74	72	77	77
CX3CR1	100	83	79	74	81	81
Siglec-F	x	100	x	x	x	x
CADM1	100	96	95	91	88	97
CD26	100	85	88	87	82	84
CD11c	100	70	77	77	71	71
Siglec-H	x	100	x	x	x	x
CD304	100	93	95	94	94	95
CD163	100	71	78	77	81	82
CD169	100	73	80	76	80	80
CD115	100	75	84	80	83	83
HLA-DR B1	100	77^**^	77	83^#^	75	73
CD68	100	66	69	74	74	66
MARCO	100	67	62	65	62	63
MPO	100	86	86	79	82	88

x indicates absence of a homolog.

^**^Highest bit score BLAST results in mouse is the H2-Eb1 gene.

^#^Partial sequence match found in *Eonycteris spelaea* genome.

underlined fields indicate that the respective species’ homolog was used as the base comparator when calculating % identity.

In humans, circulating monocytes are sub-divided based on CD14 and CD16 expression^21^. All bat genomes analyzed possess homologs of CD14 (Table 1). Humans have two gene homologs for CD16 as the result of a gene duplication event - FCGR3A and FCGR3B^22^. Only one homolog of FCGR3 was observed in the bat genomes examined (Table 1). Mice also possess only one homolog of FCGR3, but it exhibits significant sequence divergence from human FCGR3A/B, and aligns more closely with FCGR2 homologs instead (Fig. S1). Bat FCGR3 was observed to cluster with human, bovine and pig FCGR3 homologs and separately from mouse FCGR3 (Fig. S1).

In mice, the F4/80 marker (encoded by ADGRE1) is widely used to identify mature macrophages^14^. The ADGRE family of receptors has three homologs in mice, ADGRE1, 4 and 5. Humans, bats and other Laurasiatherian mammals have five homologs - ADGRE1 to 5 - indicating that differences in evolutionary pressure at this locus could have also resulted in changes in receptor utilization and distribution patterns. It was also observed that Siglec-F, a mouse alveolar macrophage marker, does not have a homolog in bats (as well as in humans). On the other hand, several surface markers relevant for differentiating human macrophages from monocytes – such as CD163, CD169 and CD206 - were observed to have conserved gene homologs in bats (Table 1).

**Figure 1:**
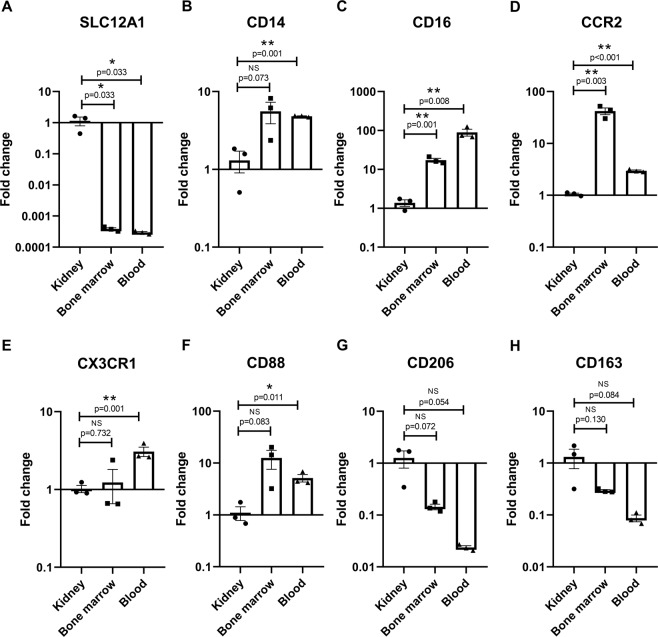
Quantification of gene expression in *E. spelaea* tissue. Transcript levels of (**A**) SLC12A1, (**B**) CD14, (**C**) CD16, (**D**) CCR2, (**E**) CX3CR1, (**F**) CD88, (**G**) CD206 and (**H)** CD163 in kidney tissue, whole blood and bone marrow cell suspensions were quantified by real-time PCR. Gene expression was normalized to kidney tissue. Data is presented as mean ± SEM from tissues derived from three individual bats. (*indicates p < 0.05, **indicates p < 0.01).

Subsequent experimental validation of surface markers was performed on tissue obtained from the cave nectar bat *E. spelaea*, as our group is presently focused on establishing this species as a model organism for dissecting bat-virus interactions via a local captive breeding colony. We first investigated the relative gene expression patterns of selected markers in *E. spelaea* kidney, bone marrow and whole blood using quantitative real-time PCR. The Na/K co-transporter SLC12A1 was used as a control in this experiment and showed the expected kidney-restricted expression (Fig. 1A). Transcript levels of CD14, CD16, CCR2, CX3CR1, and CD88 were significantly higher in blood compared to kidney tissue (Fig. 1B–F), consistent with the distribution expected if these markers were present on circulating monocytes and granulocytes. They also had generally higher expression in the immune-cell rich bone marrow compared to kidney tissue (Fig. 1B–F). Transcripts for CD206 and CD163 on the other hand did not have a significantly higher expression in blood tissue compared to bone marrow or kidney, consistent with a predominant expression on tissue-resident macrophages for these two genes (Fig. 1G,H). In conclusion, we show that these selected markers – CD14, CD16, CCR2, CX3CR1, CD88, CD206 and CD163 – have genetic homologs in bats, are transcriptionally expressed, and have an expression pattern consistent with their expected functions. Hence these markers are good candidates for identifying cross-reactive antibodies or generating bat-specific antibodies for immunophenotyping myeloid cells in bats.

### Immunophenotyping *E. spelaea* BMDMs

Using this information, cross-reactive antibodies against some of these bat myeloid markers were identified (Table S2). In general, we favored antibodies with reported cross reactivity to at least one other Laurasiatherian mammal in addition to humans or mice, as a conservation in the binding ability across species indicates the antibody binding region is evolutionarily constrained, and that the epitope recognized is likely to be conserved in bats as well^23^.

**Figure 2:**
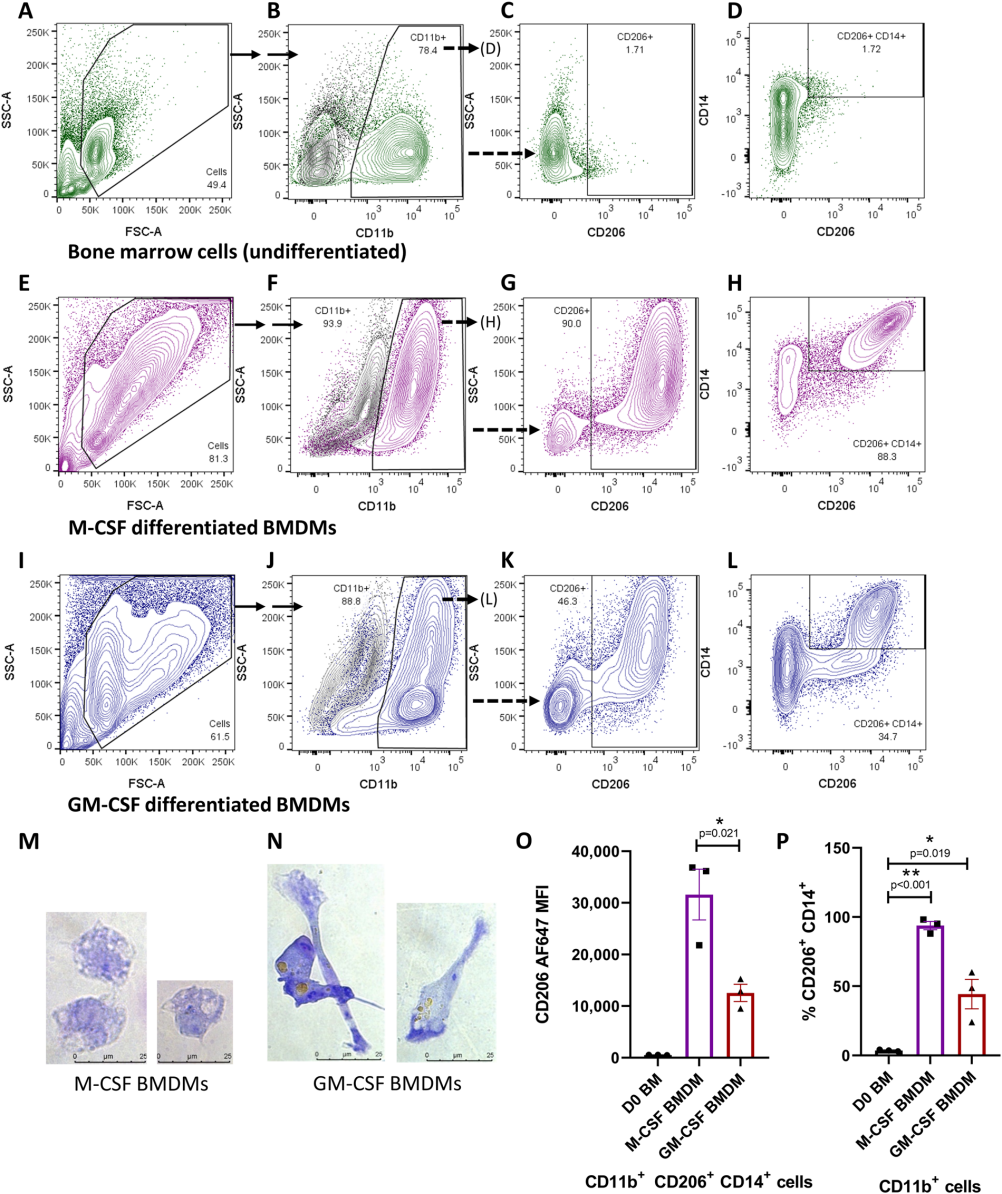
Immunophenotyping bat BMDMs. Flow cytometry analysis of undifferentiated bat bone marrow cells (**A–D**, in green), M-CSF differentiated BMDMs (**E–H**, in purple) and GM-CSF differentiated BMDMs (I-L, in blue) demonstrating changes in FSC-A/SSC-A properties (**A,E,I**), CD11b expression (**B,F,J**), CD206 expression (**C,G,K**), CD206 and CD14 co-expression (**D, H, L**) during bat macrophage differentiation. Black contour plots indicate staining intensity in CD11b BV711 FMO stained controls. (M) Micrographs of Giemsa stained CD11b^+^ CD14^+^ CD206^+^ M-CSF BMDMs, and (**N**) CD11b^+^ CD14^+^ CD206^+^ GM-CSF BMDMs. (**O**) CD206 staining intensity in CD11b^+^ CD206^+^ CD14^+^ cells in bone marrow cells, and M-CSF and GM-CSF BMDMs. (**P**) Percentage of CD11b^+^ cells which stain positively for both CD14 and CD206 in bone marrow cells, M-CSF and GM-CSF BMDMs. Flow cytometry data and micrographs are representative of results from three individual bat derived tissues. Data in (**O,P**) is presented as mean ± SEM from three individual bat derived tissues. Scale bars represent 25 µm.

Next, *E. spelaea* bone marrow cells were differentiated for six days in the presence of bat M-CSF containing culture medium using a similar protocol to that described for *Pteropus alecto* by our group recently^23^. The resulting adherent cells were stained with cross reactive antibodies against CD11b, CD14 and CD206, and the antibody staining profile was compared to that of undifferentiated bone marrow cells via flow cytometry. In undifferentiated *E. spelaea* bone marrow, CD11b^+^ cells comprised 78.5% of total live singlet cells (±1.1%, n = 3), indicating that the bone marrow is an important site for myeloid leukocyte production in bats as in other mammals (Fig. 2A,B). Differentiation in the presence of M-CSF cloned from *E. spelaea* resulted in an increase in the size and granularity of CD11b^+^ cells, and prominent expression of the CD206 surface marker (Fig. 2E–G). Undifferentiated bone marrow cells did not stain strongly for CD206 (Fig. 2C,D), while 93.8 ± 5.2% of CD11b^+^ cells were CD206^+^ after M-CSF differentiation (n = 3, Fig. 2G,P). Importantly, all of the CD206^+^ SSC^hi^ cells resulting from differentiation also stained strongly for the CD14 surface marker (Fig. 2H vs 2D). Giemsa staining of sorted CD11b^+^ CD206^+^ cells revealed large, irregular shaped cells with prominent vacuoles, consistent with the morphology of BMDMs (Fig. 2M). It was also observed that a variable fraction of the adherent cells derived six days after M-CSF differentiation were CD11b^−^ and had a fibroblast morphology (data not shown). Although the exact ontology and functional significance of these cells are presently unclear, we show that these CD11b^−^ cells are also entirely negative for CD14 and CD206 (Fig. S2), highlighting the specificity of CD14 and CD206 staining to the BMDM population.

Differentiation of bone marrow cells in the presence of GM-CSF also gives rise to a monocyte-derived macrophage population (along with dendritic cells)^24^. To explore whether the CD206 surface marker is highly expressed on GM-CSF differentiated bat macrophages as well, *E. spelaea* bone marrow cells were differentiated in the presence of GM-CSF derived from the closely related pteropodid bat *P. alecto*^23^. A distinct population of large, adherent CD11b^+^ SSC^hi^ cells arose after GM-CSF differentiation co-stained strongly for both CD206 and CD14, indicating a macrophage phenotype (Fig. 2I–L). Similarly, sorted CD11b^+^ CD206^+^ cells displayed a macrophage morphology after Giemsa staining and imaging (Fig. 2N). Compared to M-CSF differentiated macrophages, pseudopodia-like structures were observed more frequently in the GM-CSF differentiated macrophages. Internalized debris or particular matter was visible within the vacuoles of some macrophages (Fig. 2N). It should be noted that although both M-CSF and GM-CSF BMDMs expressed CD206, the staining intensity for CD206 antibody was higher in M-CSF BMDMs (Fig. 2O). GM-CSF differentiation resulted in a more heterogeneous population of adherent myeloid cells compared to M-CSF, consistent with literature reports in other mammals^24^. Out of the resulting CD11b^+^ adherent cells upon GM-CSF differentiation, 55.7 ± 5.2% stained negatively for the macrophage markers CD14 and CD206 (n = 3, Fig. 2L,P). In conclusion, we demonstrate that CD206 is strongly expressed on both M-CSF and GM-CSF differentiated bat BMDMs, and that bat macrophages co-stain with three independent myeloid markers, CD11b, CD14 and CD206.

### Immunophenotyping circulating monocytes and granulocytes in *E. spelaea*

**Figure 3:**
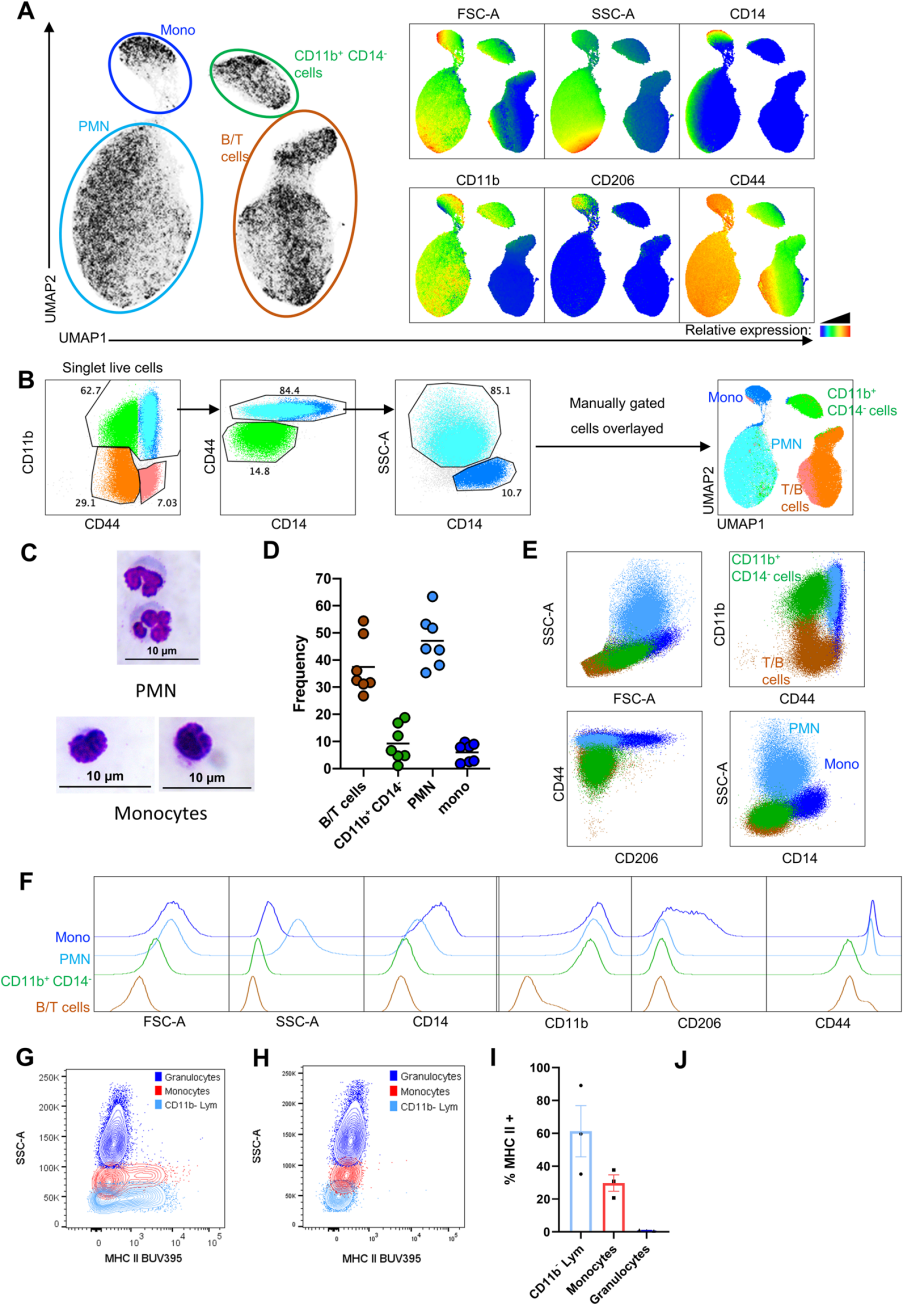
Immunophenotyping circulating bat monocytes and PMNs. (**A**) Singlet, live, blood leukocytes from seven animals were subjected to unsupervised analysis by the UMAP dimension reduction algorithm, and then concatenated. Subsets corresponding to monocytes (blue), PMNs (cyan), putative NK cells (green) and B/T lymphocytes (brown) were identified based on the expression heat maps of FSC-A, SSC-A, CD14, CD11b, CD206 and CD44 (**B**) Manual gating strategy for identifying immune subsets in blood leukocytes (left panels), including monocytes, PMNs, putative NK cells and B/T cells, that were then overlayed onto the UMAP plot (right panel), demonstrating that this gating strategy is capable of identifying the discrete subsets observed via unsupervised analysis. (**C**) Micrographs of Giemsa stained PMNs and monocytes. Images are representative of results from three individual bat derived tissues. (**D**) Percentage of major immune subsets in the total leukocyte population (n = 7 individual bat derived tissues). (**E,F**) The relative phenotype of PMNs, monocytes, B/T cells and putative NK cells are shown by back-gating cells defined in the UMAP space depicted in (**A**) in (**E**) SSC-A vs FSC-A, CD44 vs CD11b, CD206 vs CD14 and CD14 vs SSC-A plots, or as (**F**) Histograms of FSC-A and SSC-A parameters, and CD14, CD11b, CD206 and CD44 cell-surface expression on the indicated immune cell populations. (**G,H**) MHC-II staining intensity in granulocytes, monocytes and CD11b^-^ lymphocytes from one representative bat tissue. (**H**) represents a fully stained *E. spelaea* sample with no MHC-II staining across all cell populations. (I) Percentage of MHC-II^+^ cells in granulocytes, monocytes and CD11b^-^ lymphocytes. Only *E. spelaea* individuals demonstrating MHC-II binding to their cells were included in this analysis (n = 3 individual bat derived tissues). Scale bars represent 25 µm.

Peripheral blood was obtained via venipuncture of the cephalic vein running along the antebrachial membrane of the bat wing^25^. Approximately 50–80 µl of blood was routinely obtained per bat, yielding on average 50,000–80,000 total leukocytes for flow cytometry analysis after RBC lysis. Cells were stained with identified cross-reactive antibodies against CD44, CD11b, CD206 and CD14. We first performed an unsupervised analysis of the flow cytometry data using the UMAP (uniform manifold approximation and projection) nonlinear dimensionality-reduction technique^26^. Distinct clusters corresponding to monocytes, granulocytes, putative NK cells, and B/T lymphocytes were observed (Fig. 3A). Based on the results of this unsupervised analysis, a sequential gating strategy was established for circulating PMNs and monocytes. PMNs were identified as CD44^hi^ SSC^hi^ cells within the CD11b^+^ fraction (Fig. 3B), and displayed a polymorphonuclear structure upon imaging (Fig. 3C). Monocytes were identified as CD11b^+^ CD44^hi^ SSC^mid^ CD14^+^ cells (Fig. 3B). Sorted monocytes displayed the expected mononuclear morphology (Fig. 3C). Granulocytes accounted for approximately 45% of total leukocytes in circulating *E. spelaea* blood (Fig. 3D), resembling the circulating granulocyte proportion in human leukocytes^27,28^. In contrast, circulating granulocytes constitute ~15% of total leukocytes in mice when averaged across an extensive panel of different strains^29^. Monocytes accounted for ~5% of total bat blood leukocytes (Fig. 3D), consistent with literature reports for humans^27,28^. Some bat monocytes were observed to stain positively for the mannose receptor, CD206, albeit weakly when compared to differentiated macrophages (Fig. 3E,F). CD206 expression was specific for monocytes and was not observed on the other major blood leukocyte populations defined here (Fig. 3E,F). A subset of circulating bat monocytes expressing the typically macrophage-restricted mannose receptor is intriguing, and the physiological relevance of this observation is presently under further investigation.

Leukocyte MHC-II expression was also assessed using the rat monoclonal antibody 2G9, which recognizes the I-A/E^d^ alloantigens of the MHC class II complex^30^. PMNs did not exhibit significant MHC-II expression (Fig. 3G), as they are not expected to possess antigen-presenting function. Approximately 30% of monocytes stained positive for the 2G9 antibody (Fig. 3G,I). It was also observed that CD206^+^ monocytes had a higher MHC II^+^ proportion compared to CD206^−^ monocytes (Fig. S3). A significant fraction of CD11b^−^ lymphocytes also showed binding to the 2G9 antibody (Fig. 3G). This population is likely to include both B and T cells, as we previously reported that MHC-II staining is observed on B cells as well as on a fraction of CD3^+^ T cells in the closely related fruit bat *P. alecto*^31^. CD3^+^ cells were identified by intracellular staining with the CD3–12 monoclonal antibody, which detects a highly conserved epitope on the cytoplasmic domain of CD3ε^31^. A fraction of human, but not mouse, T cells express MHC-II^32^. Similarly, we observed that a variable fraction of *E. spelaea* CD3^+^ T cells express MHC-II (Fig. S4), another parallel with humans. Interestingly, 4/7 bat leukocyte samples had absent 2G9 staining across all cell types (Fig. 3H). This is most likely due to polymorphisms at the I-A/E sub-regions resulting in varying combinations of cell-surface expressed alloantigens amongst different *E. spelaea* specimens in our colony.

Lastly, myeloperoxidase (MPO) expression in leukocytes was investigated. CD14 staining intensity and granularity was used to differentiate between granulocytes, monocytes and lymphocytes in fixed, permeabilised and stained cells (Fig. S5A). Consistent with MPO storage in pre-formed granules within myeloid cells of other species, MPO staining was highest in bat granulocytes, followed by monocytes (Fig. S5B–D). Cross-reactive antibodies against MPO would also be a useful marker in histological studies to verify the presence or absence of granulocyte infiltration in various tissues.

### Immunophenotyping myeloid cell populations in *E. spelaea* lung

**Figure 4:**
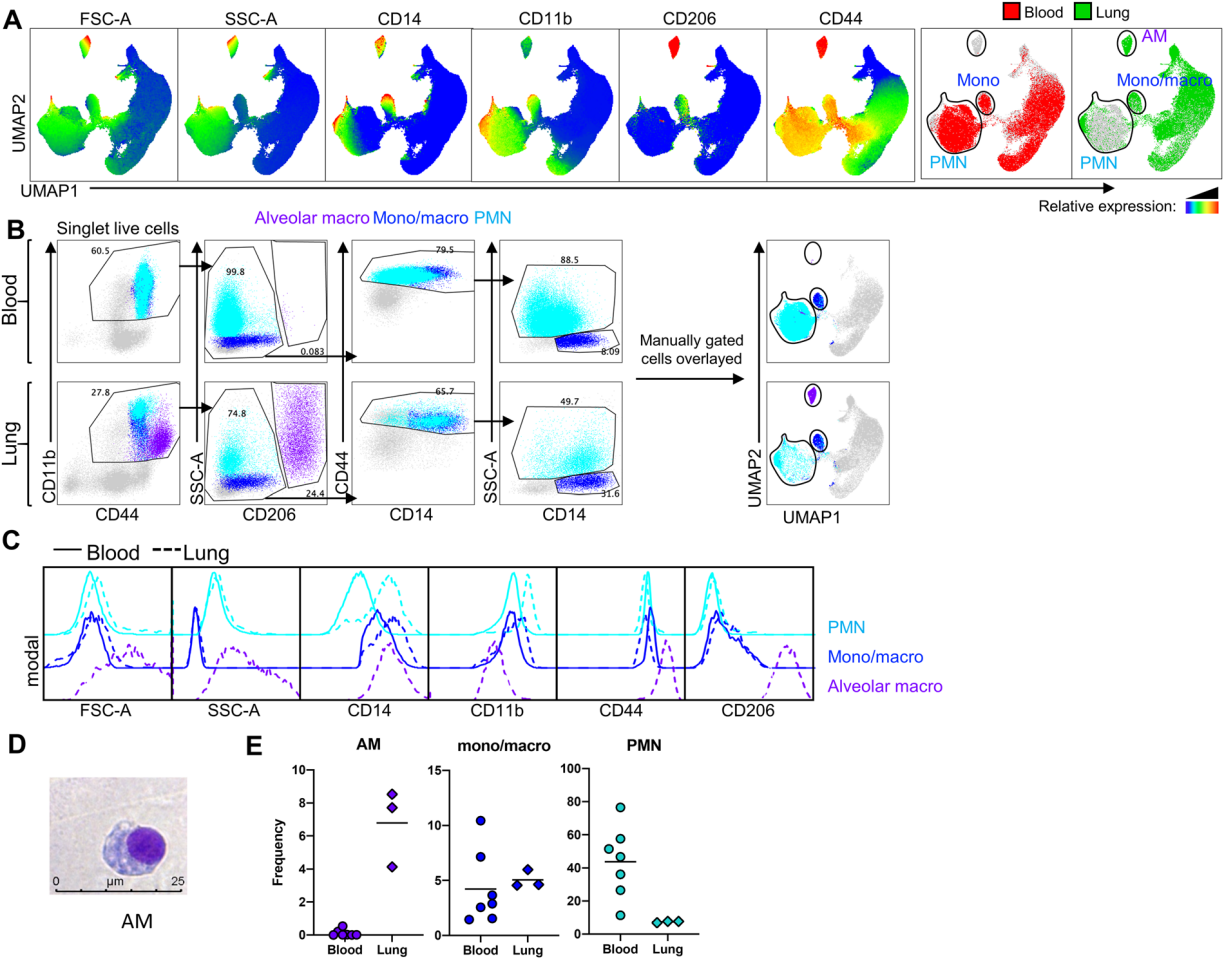
Immunophenotyping bat lung myeloid cells. (**A**) Singlet, live cells from blood leukocytes (n = 7 individual bat derived tissues) and lung tissue (n = 3 individual bat derived tissues) were concurrently subjected to unsupervised analysis by the UMAP algorithm, and then concatenated into a single plot. Based on the expression heat maps of FSC-A, SSC-A, CD14, CD11b, CD206 and CD44, subsets corresponding to monocytes and PMNs were identified in the blood, and subsets corresponding to AMs, mono/mac, and PMNs were identified in the lung. (**B**) Manual gating strategy for identifying each of these myeloid cell populations in blood and lung leukocytes. The manually gated subsets were then overlayed onto the concatenated UMAP plot. (**C**) Histograms of FSC-A and SSC-A parameters, and CD14, CD11b, CD206 and CD44 cell-surface expression on the indicated immune cell populations in blood leukocytes and lung tissue are shown. (**D**) Micrographs of Giemsa stained alveolar macrophage. Images are representative of results from three individual bat derived tissues. Scale bar represents 25 µm. (**E**) Percentage of the indicated myeloid cell types in the CD44^+^ population in blood leukocytes and lung cells.

We next examined myeloid cell populations in the lung, an organ of special interest in bats as they are reservoir hosts to several important respiratory pathogens. Stained leukocytes from blood were reacquired with the same voltage parameters used for lung cell analysis, to enable a direct comparison of myeloid populations between the two tissues. Unsupervised UMAP analysis was performed simultaneously on stained lung and blood samples. Lung cells exhibited a unique CD206^hi^ population with very high granularity (Fig. 4A,B), which corresponded to AMs described in human and macaque lung tissue^33,34^. Two clusters were also observed in lung cells which corresponded well to the previously identified granulocyte (PMN) and monocyte populations in blood leukocytes, although the lung monocyte-like population is likely to have a greater heterogeneity than blood monocytes (Fig. 4A,B). This information enabled a gating strategy to identify these three populations in lung tissue (Fig. 4B). Lung PMNs had similar SSC/FSC characteristics to circulating PMNs (Fig. 4A,C), while the lung AM population had a significantly higher SSC than either granulocyte population (Fig. 4A,C). Sorted AMs were also observed to be larger than granulocytes or monocytes, with prominent vacuoles within the cytoplasmic space (Fig. 4D). Despite almost identical SSC/FSC parameters, lung PMNs displayed a higher expression of CD11b and CD14 compared to circulating PMNs (Fig. 4C). This is consistent with observations in human neutrophils which have been reported to switch to an activated phenotype after homing to the lung, even under steady-state conditions^35^.

Of the different myeloid populations, CD206 expression was highest in the bat AMs (Fig. 4C), even after accounting for the higher auto-fluorescence in AMs (Fig. S6A). CD206 staining on AMs resembled the intense staining previously observed for BMDMs. Lung monocytes/ monocyte-derived cells exhibited a weak staining for CD206, similar to circulating monocytes (Figs. 4C, S6B). Bat AMs were CD11b^+^, although expressing this marker at a lower intensity than monocytes or granulocytes, particularly when compared to the FMO control (Figs. 4C, S6C,D). Bat AMs were also CD14^dim^, with only a minor difference in the staining intensity between AMs and the FMO control, unlike lung monocyte/monocyte-derived cells which displayed a clear CD14 signal above that of the FMO control (Figs. 4C, S6E,F). This immunophenotype of bat AMs is consistent with human and macaque AMs, which exhibit a similarly low but positive expression of CD11b, and are defined as CD14b^−^ or CD14^dim^ in the literature^34,36^.

The monocyte-like population in lungs (CD11b^+^ CD14^+^ SSC^mid^ cells) had similar size and granularity characteristics to blood monocytes (Fig. 4A,C). However, additional markers are required to fully describe this population, as even in steady-state lungs, this likely represents a heterogeneous population of infiltrating monocytes and monocyte-derived cells, including interstitial macrophages. All three lung myeloid cell types comprised approximately 5–10% of total lung CD44^+^ cells (Fig. 4E).

### Gene expression changes during bat macrophage differentiation

**Figure 5:**
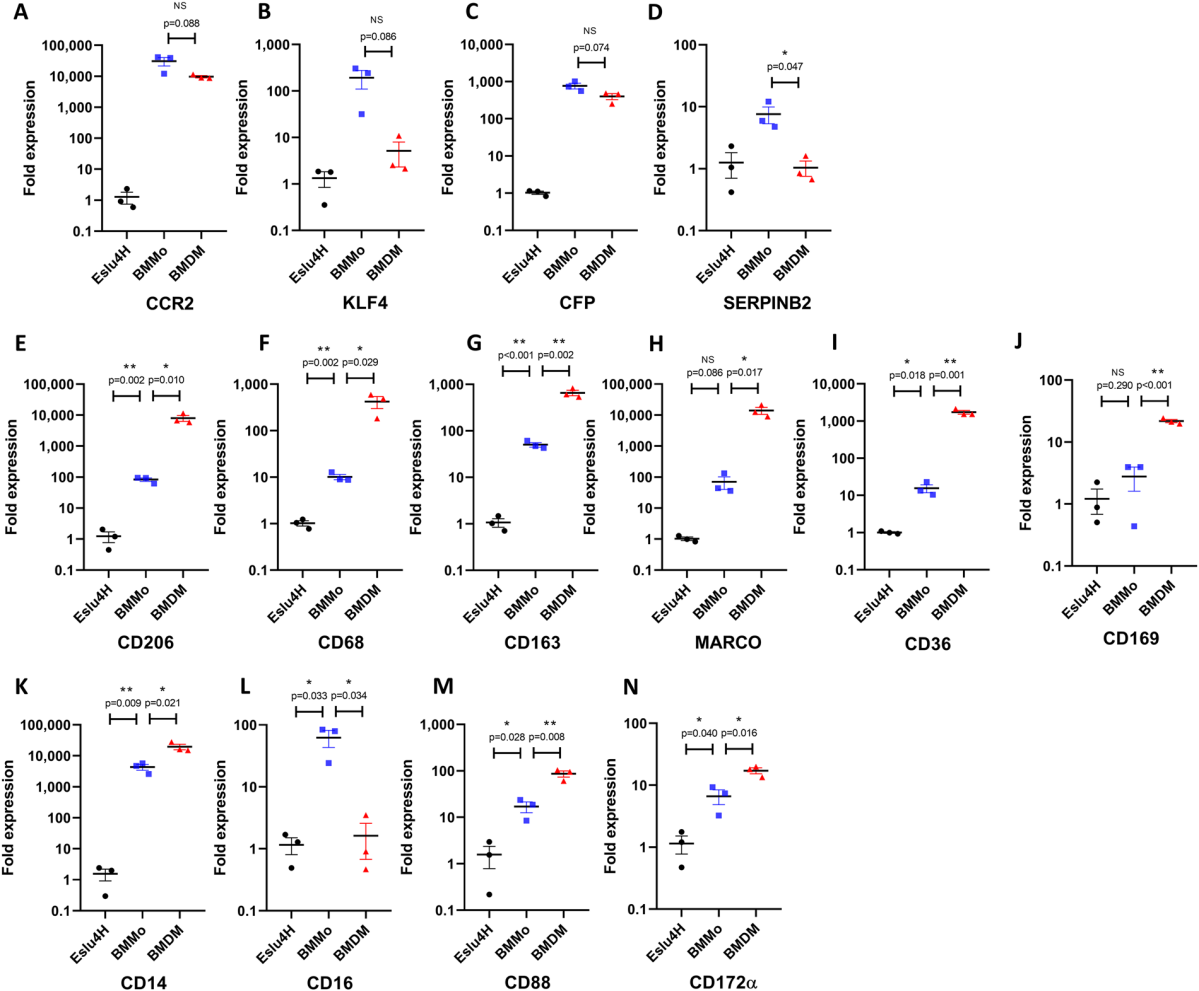
Quantification of gene expression in EsLu4H, bone marrow monocytes and BMDMs. (**A–N**) Transcript levels of the indicated target genes in EsLu4H lung cells, bone marrow monocytes, and M-CSF BMDMs were quantified by real-time PCR. Gene expression was normalized to the EsLU4H cell line. Data is presented as mean ± SEM from three individual bat derived tissues.

To identify additional myeloid markers which could be useful for immunophenotyping bat phagocytes in greater detail, we examined gene expression changes associated with bat monocyte-to-macrophage differentiation. Bone marrow monocytes (BMMos, CD11b^+^ CD14^+^ SSC^mid^) and M-CSF differentiated BMDMs (CD11b^+^ CD206^+^) were isolated by cell sorting, and transcript levels of various genes measured by real-time PCR. A lung fibroblast cell line, EsLu4H was used as a non-immune cell reference for normalization. We first validated that the monocytes and macrophages had a gene expression profile consistent with their differentiation status. The chemokine receptor CCR2, complement factor properdin (CFP), transcription factor KLF4 and serine protease inhibitor SerpinB2 were reported to be down-regulated in human macrophages upon differentiation from monocytes. Similarly, we observed that these four genes had a generally lower expression in bat BMDMs compared to BMMos, although only the down-regulation of SerpinB2 showed statistical significance (Fig. 5A–D).

Thereafter, the expression of a collection of myeloid markers was investigated across these three cell types (Fig. 5E–N). In full agreement with our previous flow cytometry analysis, CD206 and CD14 were potently expressed in bat BMDMs, while preserving expression but at lower levels in monocytes (Fig. 5E,K). Several scavenger receptors were observed to be highly expressed in bat BMDMs (Fig. 5F–I), consistent with M-CSF differentiated macrophages having extensive phagocytic and endocytosis capabilities. Expression levels of CD206, CD68, CD163, MARCO and CD36 in BMDMs were as comparably high as those of the house-keeping genes used for reference (Supplementary Table 1). The scavenger receptors tested were also expressed at marginally elevated levels in BMMos compared to the lung epithelial cells. Bat monocytes are likely to exhibit some degree of binding for antibodies targeting these receptors, similarly as we observed for CD206. Depending on the immunophenotyping strategy, this could be a useful attribute as the same antibody could delineate both monocyte and macrophage populations.

The sialoadhesin CD169 was expressed at elevated levels in macrophages compared to BMMos (Fig. 5J), consistent with Siglec-1 functioning as a macrophage marker in humans. CD88 (C5α receptor) and CD172α (signal-regulatory protein alpha) were two additional myeloid markers which showed increased expression in BMDMs and BMMos compared to the lung cells (Fig. 5M,N). However, their expression was not as prominent in macrophages as that of the scavenger receptors. CD16 had 62 fold higher average expression in BMMos than in lung epithelial cells and is likely to be a monocyte marker in bats as in other Laurasiatherian mammals (Fig. 5L). Unexpectedly, BMDMs had strongly reduced expression of CD16 compared to BMMos. Further validation of this observation awaits the development of specific antibodies.

Transcriptional profiling of the monocyte-to-macrophage transition also provides insights into the functional properties of these cells (Fig. S7A–G). M-CSF differentiated bat macrophages exhibited a broadly anti-inflammatory transcriptional signature. Inflammatory mediators such as TNFα, IL-1β and S100A12 were generally down-regulated, while anti-inflammatory genes IL-10 and TGFβ were up-regulated compared to monocytes. The remarkably high expression of IL-10 in bat BMDMs – 4,990 average fold higher than in lung epithelial cells, and 56 average fold higher than in monocytes – raises the possibility of constitutive cytokine secretion in these cells. The down-regulation of S100A12 was particularly prominent during bat macrophage differentiation and has been reported to be similarly suppressed during human macrophage differentiation (Fig. S7D). On the other hand, mice lack a homolog of S100A12. BMDMs and BMMos also had high expression of CYBB which codes for the major component of NADPH oxidase complex, indicating that these cells are likely competent in microbicidal ROS production (Fig. S7G).

### Functional and phylogenetic evidence of similarities between bat and human immune responses

**Figure 6:**
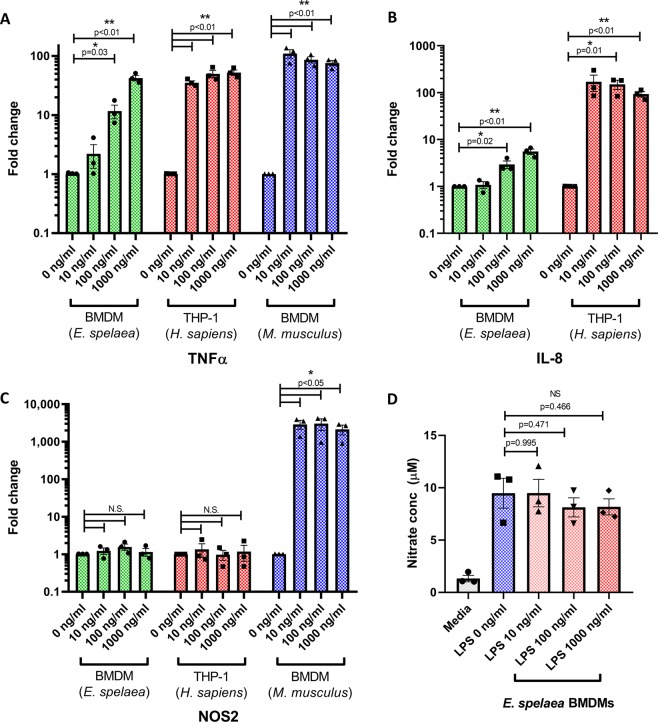
Response of macrophages to LPS stimulation. (**A–C**) Transcript levels of the indicated target genes in macrophages stimulated with LPS at the specified concentrations were quantified by real-time PCR. Gene expression was normalized to unstimulated cells. (**D**) Total nitrite/nitrate quantification from culture supernatants of *E. spelaea* M-CSF BMDMs stimulated with LPS at the indicated concentrations for 24 h. Data is presented as mean ± SEM from three individual bat derived tissues (for *E. spelaea* BMDMs), or three independent experiments (for THP-1 and mouse BMDMs).

As we had observed several instances where the immunophenotype of bat myeloid cells more closely resembled their human counterpart than mice, we further explored immune responses which have been reported to be divergent between these two species. Mice up-regulate the inducible nitric-oxide synthase NOS2 after LPS stimulation, and nitric oxide production is an important anti-microbial response in mice^37^. Although humans possess the homolog of NOS2 and can produce nitric oxide basally, a minor or no further increase in NO production has been observed with LPS stimulation^37,38^. To examine differences in the response to LPS, *Eonycteris* BMDMs, mouse BMDMs and human PMA-differentiated THP-1 cells were stimulated with LPS (Fig. 6). All three cell-types significantly up-regulated TNFα upon LPS stimulation, demonstrating the efficacy of ligand treatment (Fig. 6A). It was observed that bat macrophages had a weaker response to LPS than the human or mouse cells (Fig. 6A). Similar species-specific differences in the sensitivity to different TLR stimuli were also observed with the related fruit bat *P. alecto*^7^. Mice lack a direct homolog of the key pro-inflammatory cytokine IL-8, with the chemokines KC, MIP-2 and LIX serving as functional homologs instead^39^. Bat genomes possess a direct homolog of IL-8 (Table 1), and IL-8 was also up-regulated following LPS stimulation in *E. spelaea* BMDMs, although to a lesser extent than in in humans (Fig. 6B). Consistent with previous studies, mouse BMDMs potently up-regulated NOS2 upon LPS stimulation (Fig. 6C). However, human THP-1 cells and *Eonycteris* BMDMs failed to up-regulate NOS2 even upon stimulation with 1000 ng/ml LPS (Fig. 6F), and no further increase in total nitrite and nitrate levels were observed in the culture supernatant after stimulation of *Eonycteris* BMDMs with LPS (Fig. 6D).

**Figure 7:**
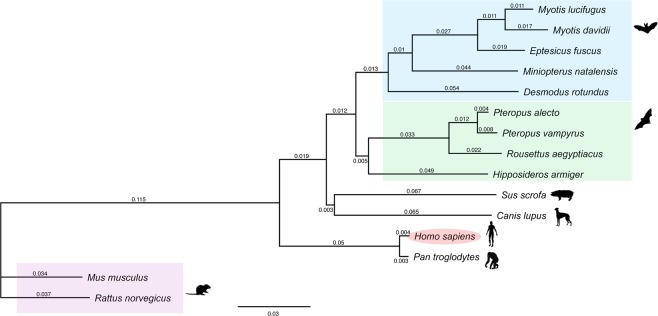
Immune-gene phylogeny across species. Maximum-likelihood tree of immune-gene orthologs in human (*Homo sapiens*), chimpanzee (*Pan troglodytes*) mouse (*Mus musculus*), rat (*Rattus norvegicus*), pig (*Sus scrofa*), dog (*Canis lupus familiaris*), little brown bat (*Myotis lucifugus*), David’s myotis (*Myotis davidii*), big brown bat (*Eptesicus fuscus*), Natal long-fingered bat *(Miniopterus natalensis*), common vampire bat (*Desmodus rotundus*), black flying fox (*Pteropus alecto*), large flying fox (*Pteropus vampyrus*), Egyptian fruit bat (*Rousettus aegyptiacus*) and great roundleaf bat (*Hipposideros armiger*) genomes. Scale bar represents 0.03 substitutions per site.

Lastly, to examine the “relatedness” of the immune systems in human, mouse and bat via an unbiased approach, we generated a phylogenetic tree of several different species using 1,723 conserved orthologs for all known immune-related genes (Figs. 7, S8). This analysis demonstrated that immune genes in bats have a closer evolutionary distance to their human orthologs than to rodent orthologs, providing support to the previous observations in our study. Interestingly, immune gene orthologs from pig (*Sus scrufus*) and the bat species included in the study were observed to share a very similar evolutionary distance to their human counterparts (Figs. 7, S8). This is a notable observation as several studies have highlighted the similarities in the immune response between pigs and humans, relative to rodents^40–43^. In conclusion, we show evidence of greater functional similarities in the immune response between bats and humans compared to mice, as well as a closer phylogenetic relationship in the immune genes of the former two species.

## Discussion

In this study, immunophenotyping approaches to identify myeloid subsets in the cave nectar bat *E. spelaea* were established. As homologs of these markers are present in the genomes of both and Yango- and Yinpterochiroptera bat lineages, the approaches utilized here are likely to be relevant for the study of other bat species as well. This enables the use of additional flow cytometry-based assays to study various immune responses such as ROS production, phagocytosis and intracellular cytokine levels in bat myeloid cells. The transcriptional and proteomic profiles of specific bat myeloid populations can also be examined in response to infection or other stimuli. Together, these investigations will provide us with a better understanding of the mechanisms responsible for viral disease tolerance in bats.

Our work also highlights approaches for expanding on this antibody panel to achieve further resolution of bat myeloid subsets. Antibodies raised against bat CD16 and CD163 would provide two key additional tools in this regard. This would be particularly useful when characterizing circulating monocyte heterogeneity. In humans, differential expression of CD14 and CD16 identify the two major circulating monocyte populations – classical (CD14^++^ CD16^−^) and non-classical (CD14^+^ CD16^++^) monocytes^44^. Bovine monocytes subsets are similarly defined based on differing CD14 and CD16 expression^45^. On the other hand, pig monocytes have been reported to display a uniform expression of CD16, with variation in CD14 and CD163 expression defining the heterogeneity instead^46,47^. We have shown that bat monocytes have a higher transcriptional expression of CD16 than lung cells, and an increase in CD16 was also observed in *E. spelaea* whole blood compared to kidney tissue. Therefore, CD16 is likely to be expressed on circulating monocytes. Whether CD16 differentiates circulating monocyte subsets as in humans and cattle or is expressed across all monocytes as in pigs remains to be seen. An anti-CD16 antibody would also enable validation of CD16 transcriptional down-regulation observed in bat BMDMs. CD163 is transcriptionally expressed in bat monocytes and is further up-regulated in BMDMs. Therefore, apart from its relevance in characterizing circulating monocyte populations, a validated bat anti-CD163 antibody could be useful for differentiating between monocytes and monocyte-derived cells in lungs and other tissues.

It was observed that the conservation of surface marker homologs at the genomic level, as well as certain functional responses showed greater similarities between bats and humans compared to mice. This may be explained at least in part by the shorter evolutionary distance to the most recent common ancestor (MRCA) between bats (and indeed the broader Laurasiatherian super-order) and primates, compared to the distance between bats and rodents^48^. Genome-wide analysis of immune genes across different mammals provided further evidence for this relationship. These observations are also consistent with previous studies that rodent genomes exhibit a faster evolutionary rate when compared to primates or pigs^49–51^. Future omics-based comparisons with representative species from these different clades would provide a more comprehensive systems-level understanding of these similarities and differences.

For now, this observation has implications for characterizing other bat immune cell subsets that are differentially described in humans and mice. This includes the innate lymphoid cells (including the natural killer cells^52^), and hematopoietic stem and progenitor cell populations^53^. This work is also relevant for comparative studies exploring the “uniqueness” of bats. Such studies ideally encompass multiple comparative species to avoid focusing on differences that are limited to a specific pairwise combination, although practical and economic considerations can affect the feasibility of such studies. In the face of practical limitations, our work suggests comparisons with humans could be more relevant than broad comparative studies with mice. Conversely, there is a significant interest in learning from bats as disease models for aging, infection and cancer resistance^2^, and a greater similarity between bats and humans would facilitate the process of translating such observations into clinically relevant interventions.

Lastly, there are limitations to our current study. We have used a rudimentary set of cross-reactive antibodies, and we haven’t extended the experimental work towards the validation of DC subsets, another important member of the MPS. This was due to restrictions placed by the lack of cross-reactive reagents, delays in generating bat-specific monoclonal antibodies, and also limitations in the quantities of bat primary tissue which makes it harder for characterizing rare immune cell subsets. At the same time, we highlight that comparative immunology studies in other non-traditional species such as dog, pig and cattle have progressed over decades with the cumulative development of reagents and strategies. We therefore expect this study to be part of a larger body of work by the bat research community to unravel the unique immunobiology of members of the order Chiroptera.

## Materials and Methods

### Identification of gene homologs in *E. spelaea*

Coding sequences (CDS) of myeloid markers or other target genes were first obtained for *Homo sapiens* or *Mus musculus* from the relevant NCBI databases. Homologs of these genes in the *E. spelaea* genome (GCA_003508835.1, assembled as described before^54^) were identified by discontiguous MegaBLAST (BLAST+ 2.7.1) with max e-value of 1e-5 and word size of 11. CDS obtained were further manually curated by comparing with the *E. spelaea* Iso-Seq transcript assembly (PRJNA427241). Homologs of human or mouse genes in the other bat species included in Table 1 were identified by a direct protein BLAST with their corresponding NCBI databases.

### Bat tissue processing

*E. spelaea* bats used in this study were part of a captive breeding colony and handled according to protocols approved by the Singhealth Institutional Animal Use and Ethics Committee (IACUC number 2015/SHS/1088). Lung tissue was obtained from n = 3 control bats sacrificed as part of a separate bat-infection protocol (Singhealth Institutional Animal Use and Ethics Committee IACUC number 2018/SHS/1385). All experiments were performed in accordance with relevant guidelines and regulations.

Bone marrow was harvested from the wing bones (humerus and radius) of sacrificed adult bats, cleaned of skin and muscle, and flushed twice with RPMI + 10% FBS (R10 media). Flushed marrow was filtered through a 100 µM cell strainer. Clumps retained on the strainer were gently dissociated with a 3 ml syringe plunger and then flushed with additional R10 media. Cells were pelleted, RBC lysed using RBC lysis buffer (eBioscience), and washed twice in R10 media before cell counting. Aliquots of 10 million cells/vial were prepared in freezing media (90% FBS, 10% DMSO) and stored in liquid nitrogen. Lung cell suspensions were prepared by cutting lung tissue into approximately 1 mm^3^ pieces using a scalpel and incubating the tissue in R10 media + 0.5% collagenase Type 4 (Worthington Biochemical Corporation) at 37 °C for 30 min with intermittent mixing. This cell suspension was passed through a 100 µM cell strainer, and any undigested tissue retentate was gently dissociated with a 3 ml syringe plunger. Cells were pelleted, RBC lysed, re-filtered through a 100 µM cell strainer, washed twice in R10 media before cell counting and aliquots stored in liquid nitrogen. Blood was obtained by venipuncture, and directly aliquoted into Eppendorf tubes containing EDTA. Total leukocytes were enriched by two rounds of RBC lysis, washed twice in R10 media and then used directly for flow cytometry staining experiments.

### Preparation of bat M-CSF and GM-CSF conditioned media

*E. spelaea* cDNA from spleen tissue RNA was generated using the OmniScript RT kit (Qiagen) according to manufacturer’s instructions. The M-CSF gene without stop codon and with restriction enzyme overhangs was amplified using the forward primer TAAGCAACCGGTCACCATGACCGCACGGGGCG and reverse primer ATTACTGCGGCCGCCACTGGCAGTTCCGCCTG using Q5 High-fidelity DNA polymerase (New England Biolabs, NEB). The resulting fragment was digested with Age1 and Not1 restriction enzymes (NEB), and ligated with similarly digested pQCXIH-mCitrine plasmid, using T4 DNA ligase (NEB). Plasmid containing the resulting C-terminal mCitrine tagged M-CSF was prepared in bulk using an endotoxin-free plasmid maxi prep kit (Omega), and then transfected into HEK293T cells with Fugene 6 transfection reagent (Promega) in Opti-MEM media (ThermoFisher Scientific). Media was replaced with complete DMEM media 6–8 h after transfection. Supernatant was harvested 48 h after transfection, cell-debris removed by brief centrifugation, passed through a 0.2 µm filter, and aliquots stored at −80 °C. Bat GM-CSF containing media was generated using a *P. alecto* GM-CSF expression construct as described previously^23^. *P. alecto* GM-CSF sequence shared 90% amino acid identity with *E. spelaea* GM-CSF.

### Bat macrophage differentiation

Frozen bone marrow cells were thawed, washed twice in R10 media and re-suspended in R10 + 10% M-CSF conditioned media (or 10% GM-CSF conditioned media). Cells were seeded at an approximate density of 2.5 million cells/well, in 2 ml of total media per well, in 6-well tissue culture treated plates. Primocin (InvivoGen) was added at a concentration of 100 µg/ml after 2 h of antibiotic-free recovery at 37 °C. Media was replaced with fresh R10 media + 10% M-CSF conditioned media + primocin on day 2 (48 h) and day 4 (96 h). On each occasion, the cell supernatant was centrifuged at 400 g for 5 min, and the cell pellet retained to ensure non-adherent/partially adherent cells are not removed prematurely. On day 6 (144 h), non-adherent cells were gently removed and media replaced with 2 ml of R10 media + primocin. After 24 h of further incubation in the absence of M-CSF/ GM-CSF, non-adherent cells were removed by discarding the supernatant from each well. Each well was washed 2x in PBS (PBS washes were collected), and cells dissociated by incubating with 0.25% trypsin for 2–5 minutes at 37 °C. Cells were further detached by gently pipetting up and down, and then washed twice in R10 media. Viable cells were counted and used for flow cytometry staining. For TLR stimulation, adherent cells were detached and enumerated on day 6, and then seeded overnight in R10 media at the appropriate density and plate format, and stimulations carried out the following day.

### Mouse BMDM and Human THP-1 differentiation

Bone marrow was flushed from the tibia and femur of adult C57BL/6 wild-type mice, RBC lysed, and washed twice in R10 media before cell counting. Cells were seeded at a density of 5 × 10^6^ cells per 10 cm tissue-culture treated dish, in R10 media + primocin + recombinant mouse M-CSF (20 ng/ml). Media was replaced on day 2 and day 4 with fresh R10 media + primocin + recombinant mouse M-CSF. On day 6, adherent cells were detached by trypsin treatment, BMDMs enumerated and seeded overnight in R10 media in 96 well-plates at a density of 100,000 cells per well. THP-1 cells were differentiated for 24 h in R10 media + primocin + phorbol 12-myristate 13-acetate (PMA, Invivogen) at a concentration of 100 ng/ml.

### Flow cytometry

Single cell suspensions were incubated in FACS buffer (PBS + 5 mM EDTA + 5% FBS) + 5% *P. alecto* pooled serum for 15 min at 4 °C. Cells were pelleted by centrifuging at ~500 g for 3 min at 4 °C, and then incubated for 45 min at 4 °C in FACS buffer containing fluorophore conjugated antibodies at the appropriate dilutions (Table S1 for more details). Cells were washed twice in FACS buffer before flow cytometry analysis. For intracellular MPO staining, cells were incubated in Fixation/Permeabilization solution (Cytofix/Cytoperm kit, BD Biosciences) for 10 minutes at 4 °C before washing in Perm/Wash buffer and proceeding with staining according to manufacturer’s instructions. For intracellular CD3 staining, cells were fixed and permeabilized using eBioscience Intracellular Fixation & Permeabilization Buffer Set (ThermoFisher Scientific) according to manufacturer’s instructions. Flow cytometry acquisition was carried out on a BD LSRFortessa (BD Biossciences), and analysis performed using FlowJo V10 software (Tree Star Inc). Cell sorting was performed on a BD FACSAria™ III (BD Biosciences) using a 100 µm nozzle. Bone marrow monocytes were sorted as Live/Dead Aqua^−^, CD11b BV711^+^ CD14 FITC^+^ SSC^mid^, using a similar gating strategy to Fig. 3B. M-CSF differentiated BMDMs were sorted as Live/Dead Aqua^−^, CD11b BV711^+^ CD206 AF647^+^ cells, using a similar gating strategy to Fig. 2. Single stained samples or beads were used for compensation controls, and unstained and fluorescence minus one (FMO) stained samples acquired as controls. The geometric mean fluorescence intensity (MFI) was used when comparing staining intensities. Gating for positively stained cells was guided by the FMO control staining threshold, as well as by the background staining intensity of non-staining populations within the stained sample (for example the SSC^low^ population in CD14 and CD206 stained samples provide an indication of the background staining intensity for both of these antibodies, which then informs the gating position for demarcating CD14^+^ and CD206^+^ populations). Leukocytes were also stained with the following isotype controls: APC-eFluor780 Rat IgG2b, K, isotype control (eBioscience), BUV395 Mouse IgG2a, K isotype control (BD Biosciences), BV711 Rat IgG2b, K isotype control (Biolegend), APC Mouse IgG1, K isotype control (Biolegend), and PE Mouse IgG2a, K isotype control (Biolegend) (Fig. S9). Antibodies against various immune markers tested in this study which displayed no or weak staining on *E. spelaea* immune tissue are listed in Table S4.

Uniform Manifold Approximation and Projection (UMAP) analyses were carried out on live, singlet cells. UMAP version 2.4.0 was implemented in Python, but executed through the reticulate R package to interface R objects with Python. UMAP was run using 15 nearest neighbors (*nn*), a *min_dist* of 0.2 and euclidean distance^26,55^. The results obtained from the UMAP analyses were incorporated as additional parameters and converted to .fcs files, which were then loaded into FlowJo to generate heatmaps of marker expression on the reduced dimensions.

### EsLu4H cell line generation

Lung cell suspensions were immortalized with the hTert gene via lentivirus transduction as described before^56^. Immortalized cells were passaged in DMEM + 10% FBS supplemented with 1x penicillin-streptomycin, 1x sodium pyruvate, and 1x non-essential amino acids (Gibco, ThermoFisher Scientific). EsLu4H cells were adherent and presented with a fibroblast morphology.

### TLR stimulation

*E. spelaea* BMDMs, mouse BMDMs and differentiated THP-1 cells seeded at a density of 100,000 cells per well in a 96-well plate were stimulated with LPS-B5 (Invivogen) at the indicated concentrations for 3 h. RNA was extracted from adherent cells using an E.Z.N.A. Total RNA Kit I (Omega Biotek) according to manufacturer’s instructions.

### Giemsa staining and imaging

Sorted cells were placed on a cover-slip in R10 media for approximately 60 min for adhesion, fixed in 100% methanol for 10 min, air-dried and stained with modified Giemsa stain (Sigma) for 45 minutes. BMDMs and alveolar macrophages were imaged with a DMi8 inverted microscope (Leica), while monocytes and granulocytes were imaged with an Axio Lab A1 microscope (Carl Zeiss).

### Quantification of gene expression

RNA was extracted from TLR stimulated BMDMs as described above. RNA was extracted from tissues (spleen, kidney or whole blood) using an RNeasy Mini kit (Qiagen). Approximately 30 mg of spleen or kidney tissue were homogenized in 350 µl TRK lysis buffer with silica beads. For whole blood, approximately 100 µl was added to TRK lysis buffer and vortexed for 60 s. The lysates were then used for RNA extraction according to the manufacturer’s instructions. For experiments involving sorted cells, RNA was extracted from 50,000–200,000 sorted cells using an RNeasy Plus Micro Kit (Qiagen). cDNA was generated using a QuantiTect Reverse Transcription Kit (Qiagen) according to manufacturer’s instructions. Real-time PCR was performed using SensiFAST SYBR No-ROX Kit (Bioline), on a CFX96 Touch Real-Time PCR Detection System (Bio-Rad). The expression of individual *E. spelaea* genes was normalized to the geometric mean of three house-keeping genes (β actin, SNPD3 and RPL4), which we observed to have a relatively constant expression level across different *E. spelaea* tissue (kidney, bone marrow and blood). For investigating the effect of LPS treatment on gene expression in human and mouse macrophages, expression of target genes was normalized to GAPDH. Primer sequences used for real-time PCR are listed in Supplementary Table 3.

### Quantification of total nitrate and nitrite

BMDMs were seeded at a density of 500,000 cells per well in a 24 well plate in R10 media overnight. Media was replaced with 0.5 ml AIM-V medium (Thermo-Fisher Scientific) the following day, stimulated with LPS-B5 at the indicated concentrations for 24 h, and supernatant collected by centrifugation. Total nitrate and nitrite levels were quantified using the Nitrate/Nitrite Colorimetric Assay Kit (Cayman Chemical) according to manufacturer’s instructions.

### Phylogenetic comparison of immune genes

To identify immune-related genes, mammalian genes annotated with Gene Ontology term “immune system process” (GO:0002376) were retrieved from http://amigo.geneontology.org. Orthologs from two primates (Human and *P. troglodytes*), nine bats (*R. aegyptiacus, P. alecto, P. vampyrus, H. armiger, M. lucifugus, M. davidii, E. fuscus, M. natalensis, D. rotundus*), one ungulate (pig, *S. scrofa*), one carnivore (dog, *C. lupus familiaris*) and two rodents (rat and mouse) were retrieved from NCBI databases (as of 15/09/19). 1723 orthologous genes in common to the above fifteen species were identified, and their CDS were concatenated to one super gene for each species. Alignment of this sequence was generated by MAFFT^57^ and used to plot the phylogeny tree by the Maximum-Likelihood method with General-Time-Reversible (GTR) model and 1,000 Bootstrap replicates in PHYML software^58^.

### Statistical analyses

Statistical testing was carried out using the unpaired t-test, using GraphPad Prism version 8. The p-values are indicated above the relevant comparison within each figure.

## Supplementary information

Sup Figure S1-S9 Sup Tables 1-4.
