# Supplementary material for: Immunophenotyping monocytes, macrophages and granulocytes in the Pteropodid bat Eonycteris spelaea
Source: Sci Rep. 2020 Jan 15;10:309. doi: 10.1038/s41598-019-57212-1 (PMC6962400; doi:10.1038/s41598-019-57212-1)
Supplement: Supplementary file 1 — Sup Figure S1-S9 Sup Tables 1-4. [file 41598_2019_57212_MOESM1_ESM.pdf]

Immunophenotyping monocytes, macrophages and granulocytes in the Pteropodid bat *Eonycteris spelaea*.

Akshamal M. Gamage[1], Zhu Feng[1], Matae Ahn[1], Foo Jee Hiang Randy[1], Hey Ying Ying[1], Dolyce Low[1], Ian H. Mendenhal[1], Charles-Antoine Dutertre[1, 2], Wang Lin-Fa[1*]

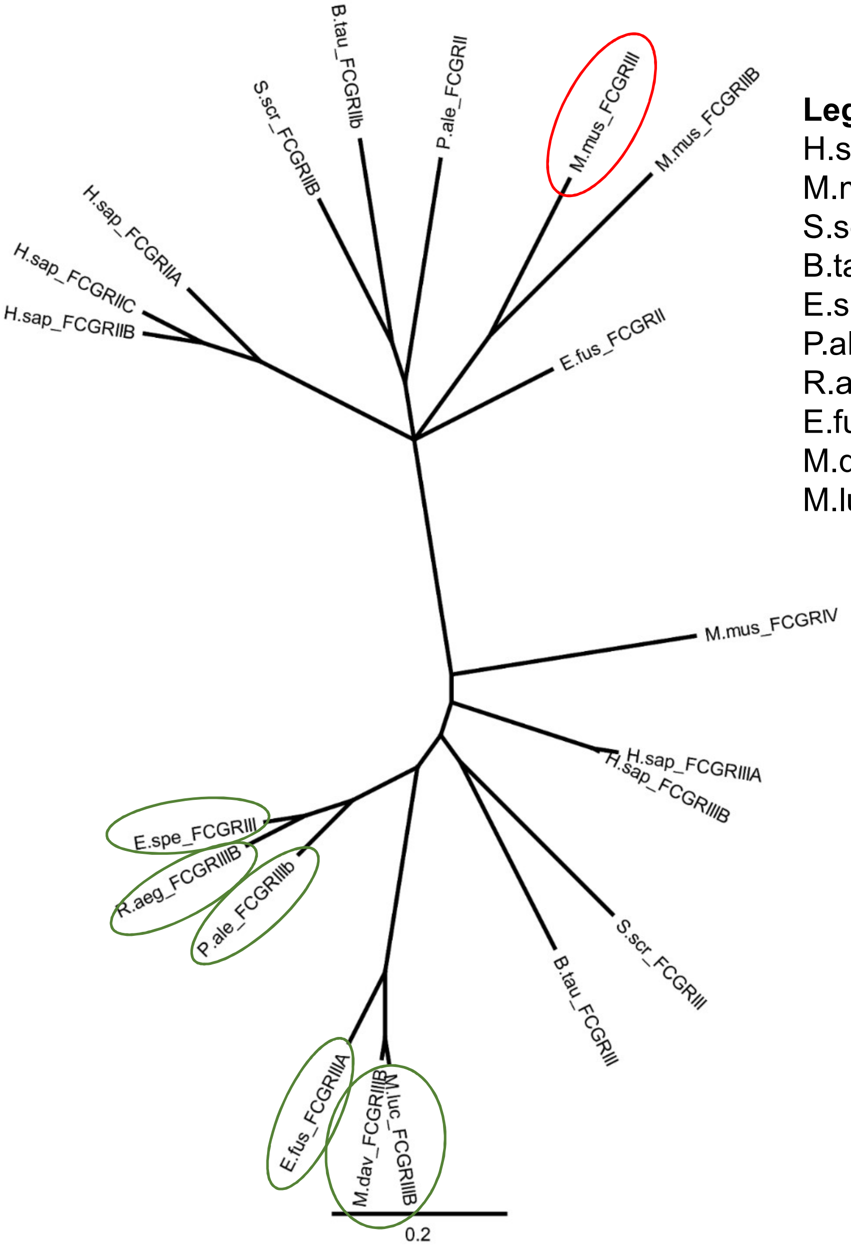

Legend
H.sap – *Homo sapiens* (human)
M.mus – *Mus musculus* (mouse)
S.scr – *Sus scrufus* (pig)
B.tau – *Bos Taurus* (cow)
E.spe – *Eonycteris spelaea* (cave nectar bat)
P.ale – *Pteropus alecto* (black flying fox)
R.aeg – *Rousettus aegyptiacus* (Egyptian fruit bat)
E.fus - *Eptesicus fuscus* (Big brown bat)
M.dav – *Myotis davidii* (David's myotis)
M.luc – *Myotis lucifugus* (Little brown bat)

Fig. S1: Neighbour joining tree for Fcγ receptor gene homologs. Bat FCGRIII sequences (in green) cluster with the human, cattle and pig FCGRIII sequences, whilst mouse FCGRIII (in red) clusters with FCGRII homologs from the other species.

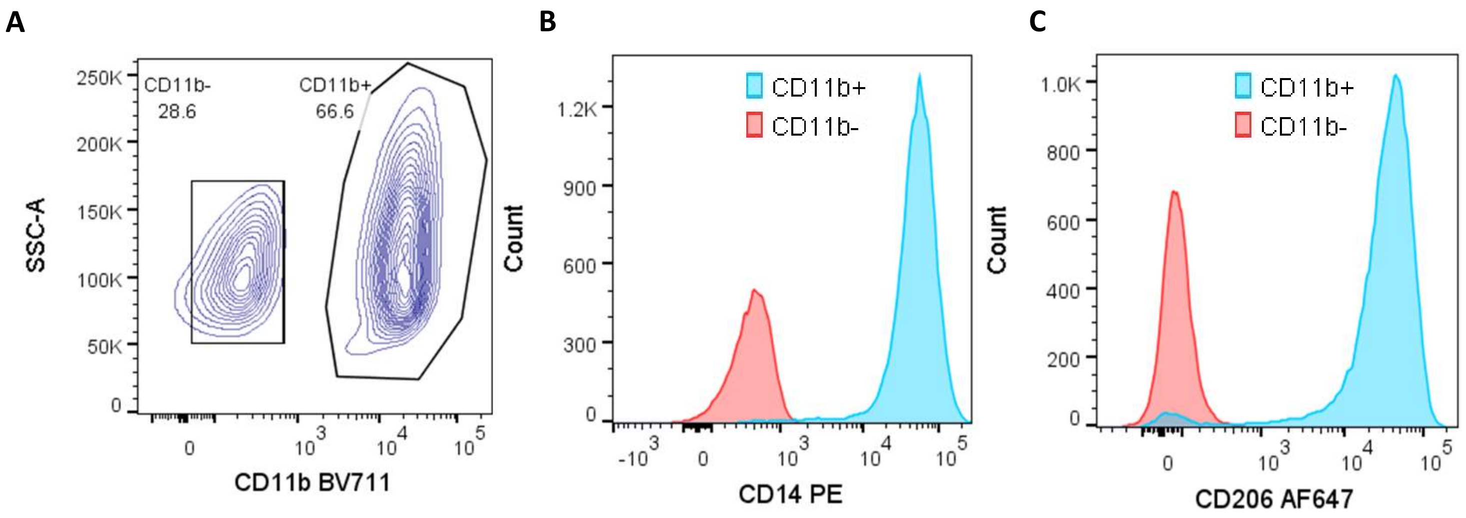

Fig. S2: CD14 and CD206 staining in CD11b + and - cell populations in CSF-1 differentiated bone marrow cells. Adherent cells obtained after 7d of M-CSF differentiation were detached, stained and analyzed by flow cytometry. After gating for single live cells, (A) $CD11b^+$ and $CD11b^-$ populations were analyzed for (B) CD14 and (C) CD206 expression. Bone marrow cells from *an E. spelaea* individual with a relatively high fraction of $CD11b^-$ cells after 7d of M-CSF differentiation were used for this analysis.

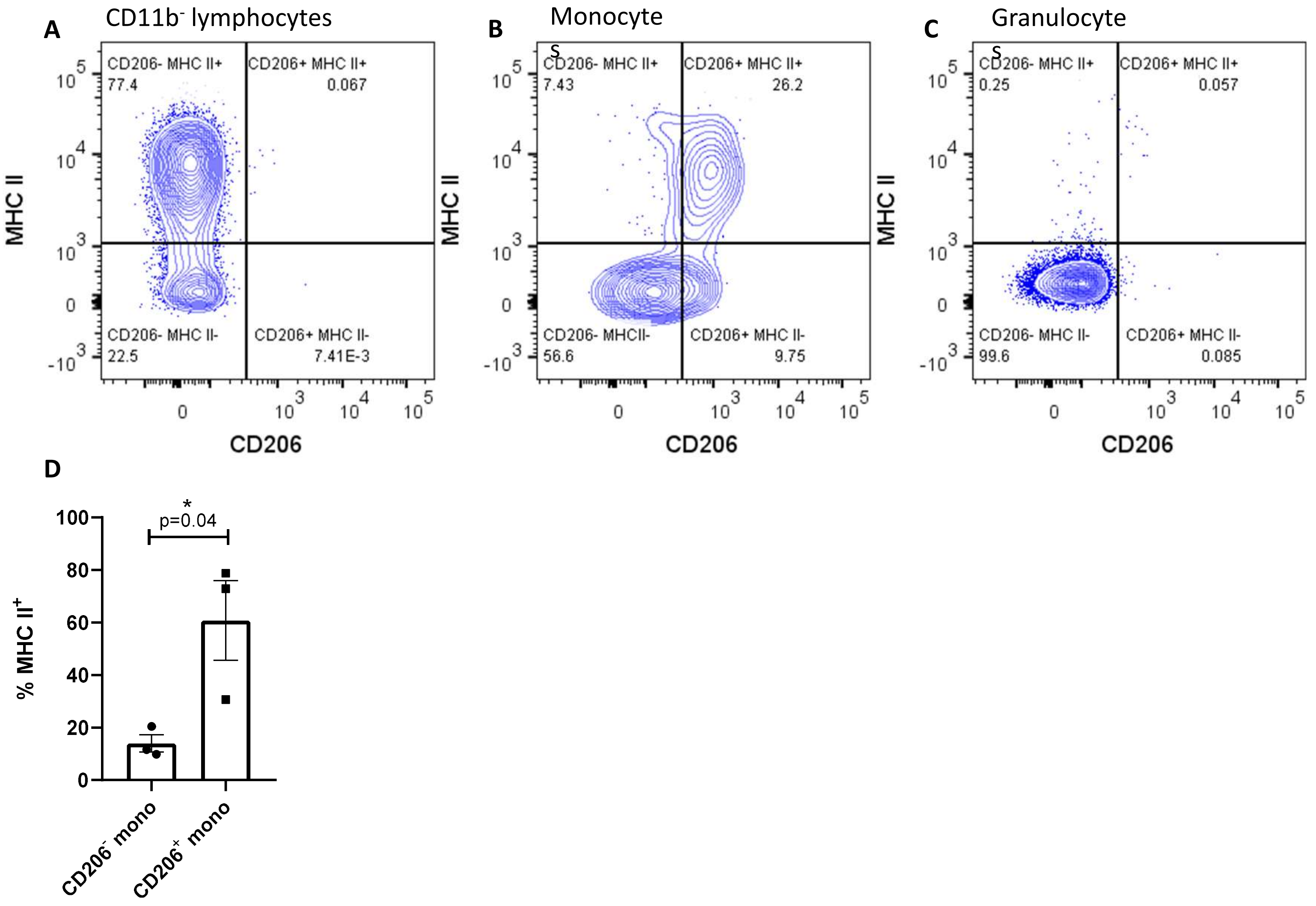

Fig. S3: MHC II vs CD206 staining in leukocyte subsets. Staining intensity of CD206 vs MHC II in (A) CD11b⁻ lymphocytes, (B) monocytes, and (C) granulocytes. Plots are representative of results from three individual bat leukocyte samples. (D) CD206⁺ monocytes have a greater MHC II⁺ fraction compared to CD206⁻ monocytes (n=3 individual bat leukocyte samples).

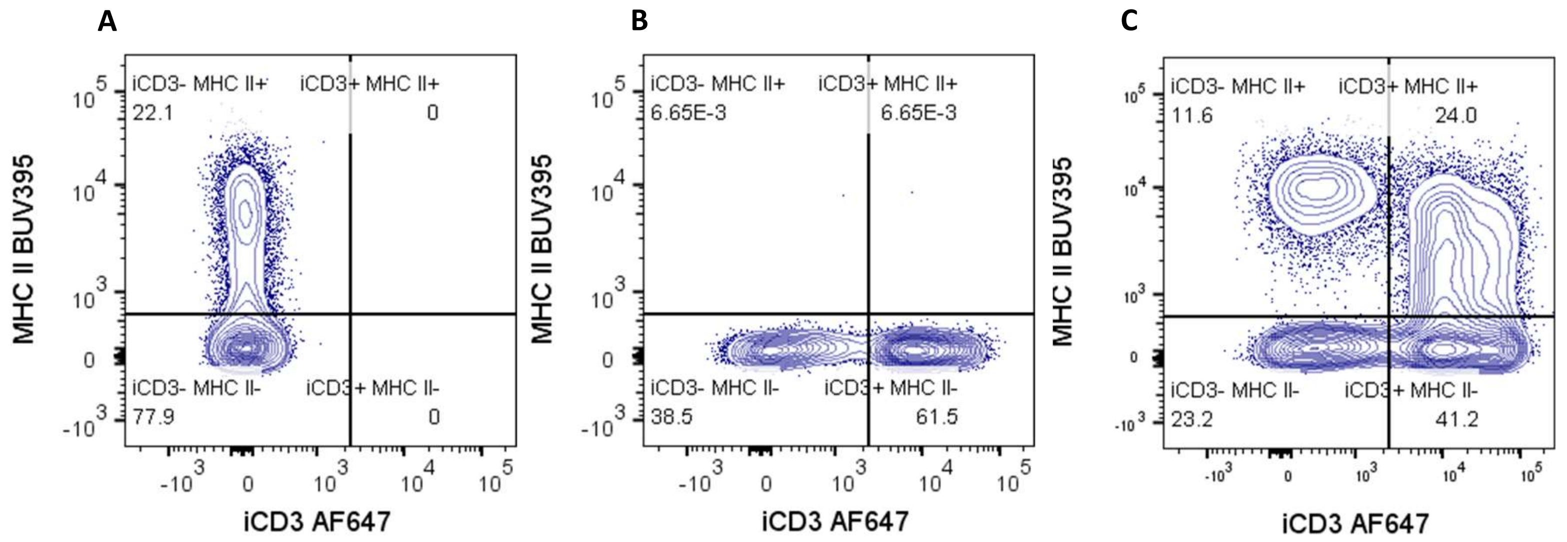

Fig. S4: MHC II staining in CD3+ cells. *E. spelaea* blood leukocytes were stained with cross-reactive antibodies for MHC II and CD3. The anti-CD3 antibody clone CD3-12 used detects an intracellular epitope of CD3 and requires permeabilisation (A) iCD3 AF647 FMO staining control, (B) MHC II BUV395 FMO staining control (C) MHC II and iCD3 stained sample, demonstrating the $iCD3^{+}$ MHC II^{+} cell population (24%)

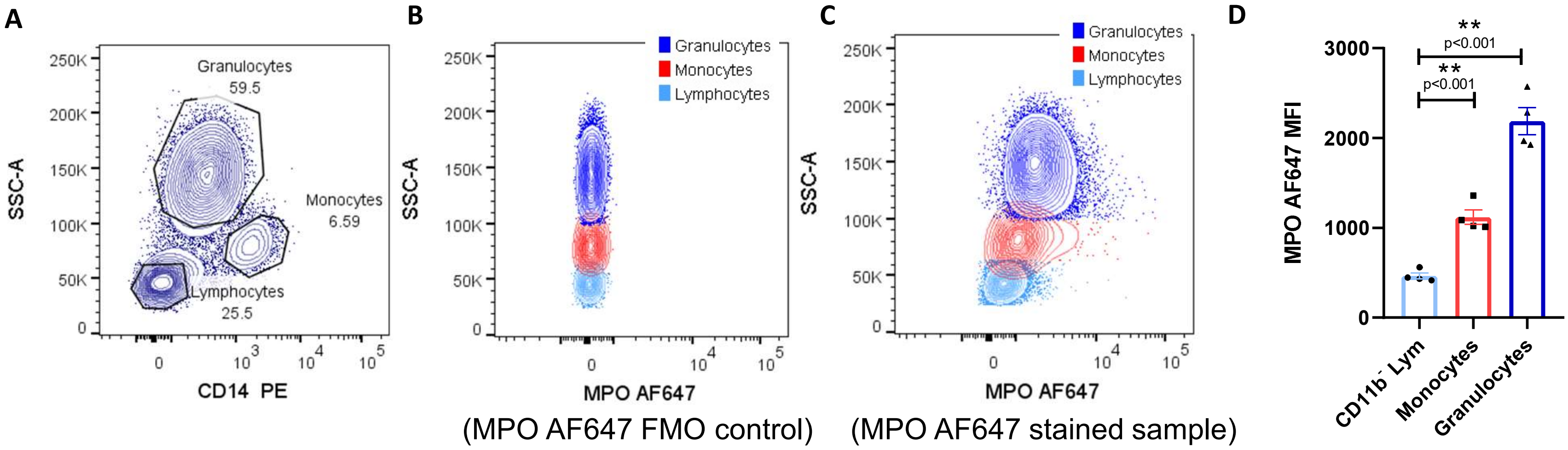

Fig. S5: Intracellular MPO staining in blood leukocytes. (A) Granulocyte, monocyte and lymphocyte populations in blood leukocytes were demarcated based on SSC and CD14 staining intensity as shown (B) MPO AF647 staining from the respective immune populations in MPO AF647 FMO stained control and (C) MPO AF647 staining from the respective immune populations in fully stained sample (D) MPO AF647 staining intensity in the indicated immune populations (n=3)

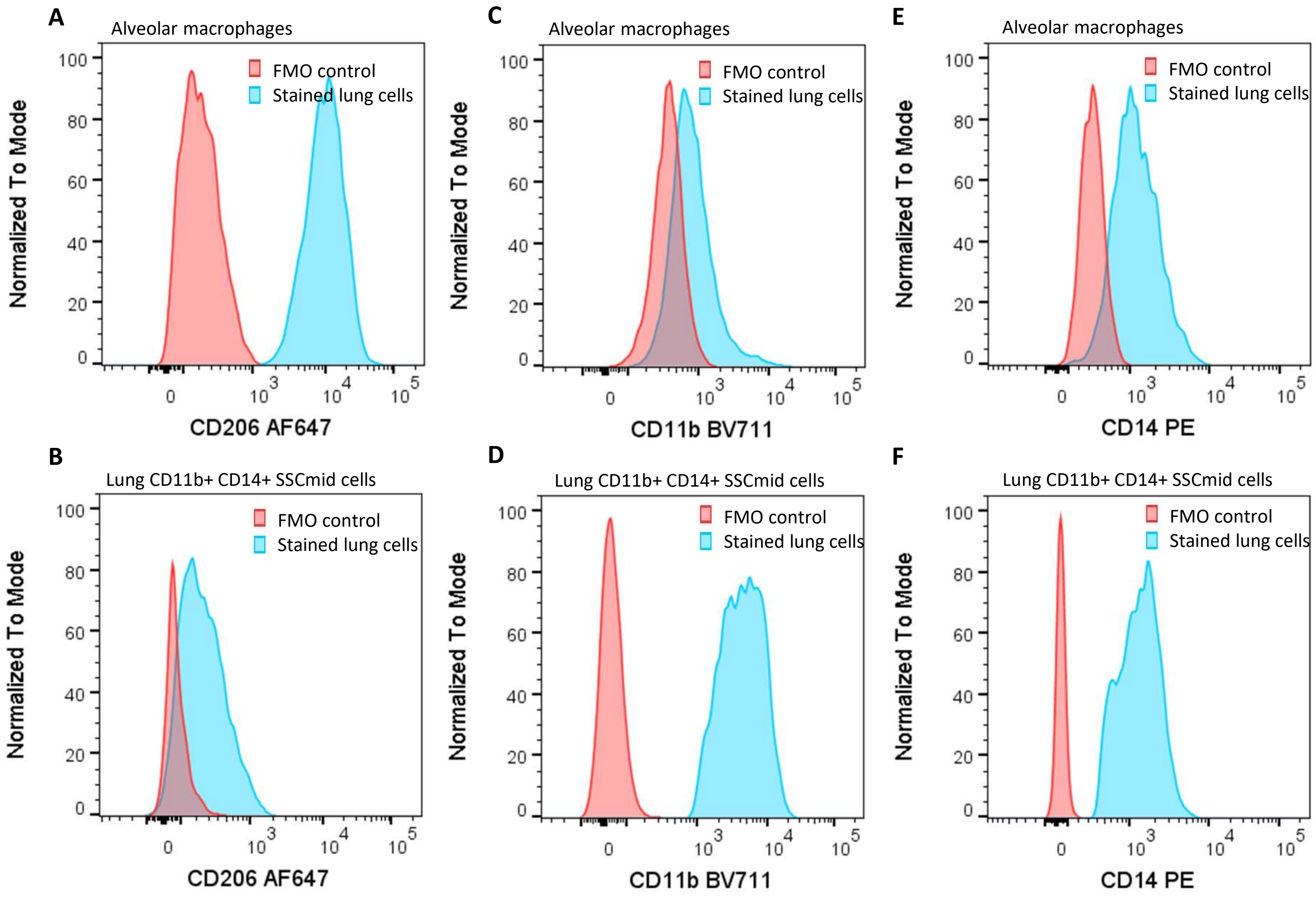

Fig. S6: Antibody staining profiles in lung myeloid cells compared to FMO staining controls. Histograms of antibody staining intensities for CD206 AF647 (A, B) CD11b BV711 (C, D) and CD14 PE (E, F) in lung alveolar macrophages (A, C, E) and monocyte/monocyte-derived cells (denoted as $CD11b^{+}$ $CD14^{+}$ SSC^{mid}) (B, D, F).

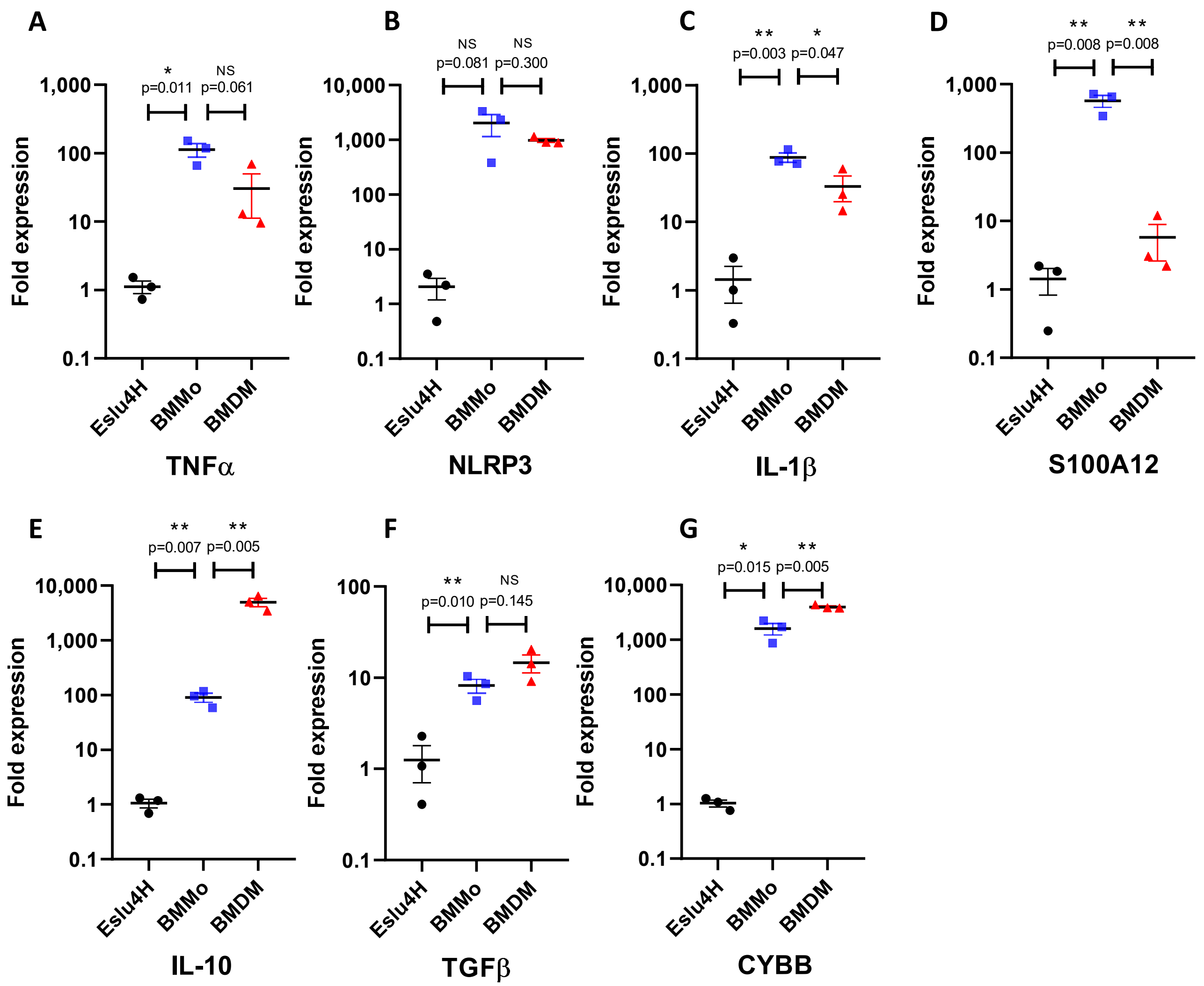

Fig. S7: Quantification of gene expression in EsLu4H, bone marrow monocytes and BMDMs. (A-G) Transcript levels of the indicated target genes in EsLu4H lung cells, bone marrow monocytes, and CSF-1 BMDMs were quantified by real-time PCR. Gene expression was normalized to the EsLU4H cell line. Data is presented as mean ± SEM from three individual bat derived tissues.

	Myotis lucifugus	*Myotis davidii*	*Eptesicus fuscus*	*Miniopterus natalensis*	*Desmodus rotundus*	*Pteropus alecto*	*Pteropus vampyrus*	*Rousettus aegyptiacus*	*Hipposideros armiger*	*Sus scrofa*	*Canis lupus*	***Homo sapiens***	*Pan troglodytes*	*Mus musculus*	*Rattus norvegicus*
Myotis lucifugus		0.029	0.042	0.093	0.113	0.128	0.131	0.133	0.128	0.155	0.154	0.158	0.158	0.254	0.257
Myotis davidii	0.029		0.048	0.099	0.12	0.134	0.138	0.139	0.134	0.162	0.16	0.164	0.164	0.26	0.263
Eptesicus fuscus	0.042	0.048		0.09	0.11	0.124	0.128	0.13	0.124	0.152	0.15	0.155	0.154	0.25	0.253
Miniopterus natalensis	0.093	0.099	0.09		0.107	0.122	0.125	0.127	0.121	0.149	0.147	0.152	0.152	0.247	0.25
Desmodus rotundus	0.113	0.12	0.11	0.107		0.122	0.125	0.127	0.122	0.149	0.148	0.152	0.152	0.248	0.251
Pteropus alecto	0.128	0.134	0.124	0.122	0.122		0.012	0.038	0.099	0.137	0.135	0.14	0.139	0.235	0.238
Pteropus vampyrus	0.131	0.138	0.128	0.125	0.125	0.012		0.041	0.102	0.141	0.139	0.143	0.143	0.239	0.242
Rousettus aegyptiacus	0.133	0.139	0.13	0.127	0.127	0.038	0.041		0.104	0.142	0.141	0.145	0.145	0.241	0.244
Hipposideros armiger	0.128	0.134	0.124	0.121	0.122	0.099	0.102	0.104		0.137	0.135	0.14	0.139	0.235	0.238
Sus scrofa	0.155	0.162	0.152	0.149	0.149	0.137	0.141	0.142	0.137		0.132	0.143	0.143	0.238	0.241
Canis lupus	0.154	0.16	0.15	0.147	0.148	0.135	0.139	0.141	0.135	0.132		0.141	0.141	0.236	0.239
Homo sapiens	**0.158**	**0.164**	**0.155**	**0.152**	**0.152**	**0.14**	**0.143**	**0.145**	**0.14**	**0.143**	**0.141**		**0.007**	**0.202**	**0.205**
Pan troglodytes	0.158	0.164	0.154	0.152	0.152	0.139	0.143	0.145	0.139	0.143	0.141	0.007		0.202	0.205
Mus musculus	0.254	0.26	0.25	0.247	0.248	0.235	0.239	0.241	0.235	0.238	0.236	0.202	0.202		0.07
Rattus norvegicus	0.257	0.263	0.253	0.25	0.251	0.238	0.242	0.244	0.238	0.241	0.239	0.205	0.205	0.07	

Fig. S8: Immune-gene phylogeny across species. Patristic distance from maximum-likelihood tree of immune-gene orthologs for each species. (Green colour indicates the closest distance, yellow indicates intermediate distance, and red indicates the longest distance between two species).

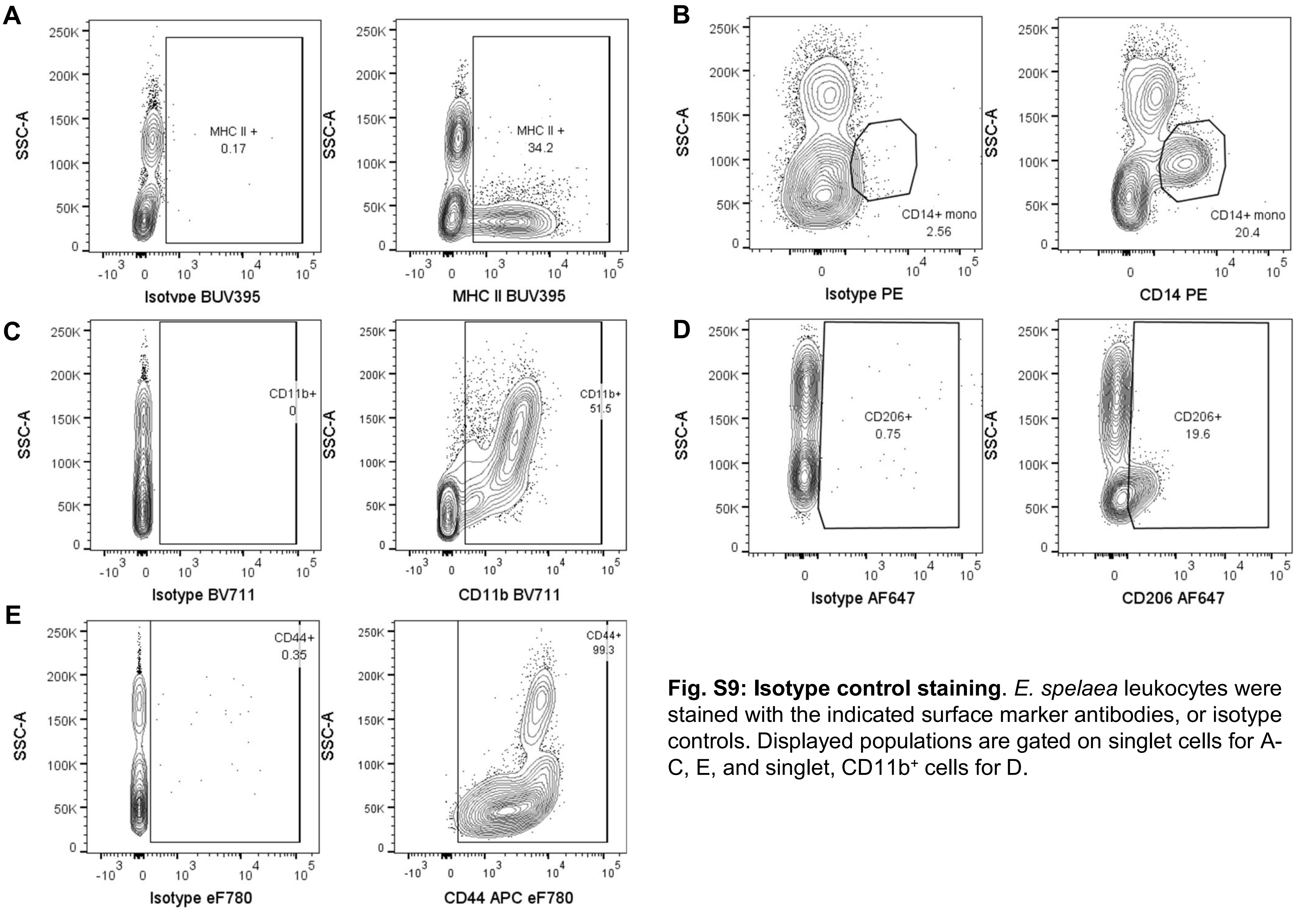

Fig. S9: Isotype control staining. *E. spelaea* leukocytes were stained with the indicated surface marker antibodies, or isotype controls. Displayed populations are gated on singlet cells for A-C, E, and singlet, CD11b^{+} cells for D.

	SNPD3	β actin	RPL4	CD206	CD68	CD163	MARCO	CD36	CD169
**Eslu4H**	20.7	17.3	17.9	32.0	25.5	28.6	31.8	30.5	29.9
**BMDM**	21.9	19.6	18.9	20.7	18.3	20.8	19.5	21.5	27.0

Supplementary Table 1: Real-time PCR Ct values for different target genes in EsLu4H cells or sorted BMDMs

Surface marker	Host species	Antibody clone	Conjugate	Source	Dilution	References for species cross-reactivity
**CD14**	Mouse, monoclonal	TUK4	PE	SantaCruz Biotechnology	1/50	Goat[1], Dog[2-3], Ferret[4], Human[5], Bovine[6]
**CD206**	Mouse, monoclonal	15-2	AF647	SantaCruz Biotechnology	1/50	Human[7], Monkey[8], Mouse[9]
**CD11b**	Rat, monoclonal	M1/70	BV711	BD Biosciences	1/400	Dog[2], Human[10], Mouse[10], Rabbit[11], *P. alecto*[12]
**CD44**	Rat, monoclonal	IM7	APC eFluor780	eBioscience	1/400	Mouse[13], Dog [14], Bovine[15], *P. alecto*[12]
**MHC II IA/E**	Rat, monoclonal	2G9	BUV395	BD Biosciences	1/400	Mouse[16], *P. alecto*[12, 17]
**CD3**	Mouse, monoclonal	CD3-12	AF647	AbDSerotec /Biorad	1/100	*P. alecto*[17], Dog [3, 18], Pig [19], Mouse [20], Chicken[21]
**MPO**	Mouse, monoclonal	2C7	AF647	Biorad	1/100	Dog[2-3], Human[22],
**Live/Dead Aqua**	-	-	-	ThermoFisher Scientific	1/400	

Supplementary Table 2: Details of cross reactive antibodies used in this study

References

1. Higgins, J. L.; Bowen, R. A.; Gonzalez-Juarrero, M., Cell mediated immune response in goats after experimental challenge with the virulent Brucella melitensis strain 16M and the reduced virulence strain Rev. 1. *Vet Immunol Immunopathol* **2018,** *202*, 74-84.
2. Novacco, M.; Comazzi, S.; Marconato, L.; Cozzi, M.; Stefanello, D.; Aresu, L.; Martini, V., Prognostic factors in canine acute leukaemias: a retrospective study. *Vet Comp Oncol* **2016,** *14* (4), 409-416.
3. Villiers, E.; Baines, S.; Law, A. M.; Mallows, V., Identification of acute myeloid leukemia in dogs using flow cytometry with myeloperoxidase, MAC387, and a canine neutrophil-specific antibody. *Vet Clin Pathol* **2006,** *35* (1), 55-71.
4. Martel, C. J.; Aasted, B., Characterization of antibodies against ferret immunoglobulins, cytokines and CD markers. *Vet Immunol Immunopathol* **2009,** *132* (2-4), 109-15.
5. Bernardo, J.; Billingslea, A. M.; Blumenthal, R. L.; Seetoo, K. F.; Simons, E. R.; Fenton, M. J., Differential responses of human mononuclear phagocytes to mycobacterial lipoarabinomannans: role of CD14 and the mannose receptor. *Infect Immun* **1998,** *66* (1), 28-35.
6. Sopp, P.; Kwong, L. S.; Howard, C. J., Identification of bovine CD14. *Vet Immunol Immunopathol* **1996,** *52* (4), 323-8.
7. Barrett-Bergshoeff, M.; Noorman, F.; Bos, R.; Rijken, D. C., Monoclonal antibodies against the human mannose receptor that inhibit the binding of tissue-type plasminogen activator. *Thromb Haemost* **1997,** *77* (4), 718-24.
8. Holder, G. E.; McGary, C. M.; Johnson, E. M.; Zheng, R.; John, V. T.; Sugimoto, C.; Kuroda, M. J.; Kim, W. K., Expression of the mannose receptor CD206 in HIV and SIV encephalitis: a phenotypic switch of brain perivascular macrophages with virus infection. *J Neuroimmune Pharmacol* **2014,** *9* (5), 716-26.
9. Leifler, K. S.; Svensson, S.; Abrahamsson, A.; Bendrik, C.; Robertson, J.; Gauldie, J.; Olsson, A. K.; Dabrosin, C., Inflammation induced by MMP-9 enhances tumor regression of experimental breast cancer. *J Immunol* **2013,** *190* (8), 4420-30.
10. Beller, D. I.; Springer, T. A.; Schreiber, R. D., Anti-Mac-1 selectively inhibits the mouse and human type three complement receptor. *J Exp Med* **1982,** *156* (4), 1000-9.
11. Rogers, C.; Edelman, E. R.; Simon, D. I., A mAb to the beta2-leukocyte integrin Mac-1 (CD11b/CD18) reduces intimal thickening after angioplasty or stent implantation in rabbits. *Proc Natl Acad Sci U S A* **1998,** *95* (17), 10134-9.
12. Zhou, P.; Chionh, Y. T.; Irac, S. E.; Ahn, M.; Jia Ng, J. H.; Fossum, E.; Bogen, B.; Ginhoux, F.; Irving, A. T.; Dutertre, C. A.; Wang, L. F., Unlocking bat immunology: establishment of Pteropus alecto bone marrow-derived dendritic cells and macrophages. *Sci Rep* **2016,** *6*, 38597.
13. Muller, C.; Zidek, L. M.; Ackermann, T.; de Jong, T.; Liu, P.; Kliche, V.; Zaini, M. A.; Kortman, G.; Harkema, L.; Verbeek, D. S.; Tuckermann, J. P.; von Maltzahn, J.; de Bruin, A.; Guryev, V.; Wang, Z. Q.; Calkhoven, C. F., Reduced expression of C/EBPbeta-LIP extends health and lifespan in mice. *Elife* **2018,** *7*.
14. Sandmaier, B. M.; Storb, R.; Bennett, K. L.; Appelbaum, F. R.; Santos, E. B., Epitope specificity of CD44 for monoclonal antibody–dependent facilitation of marrow engraftment in a canine model. *Blood* **1998,** *91* (9), 3494-3502.
15. Aguiar, D. J.; Knudson, W.; Knudson, C. B., Internalization of the hyaluronan receptor CD44 by chondrocytes. *Exp Cell Res* **1999,** *252* (2), 292-302.
16. Becker, D.; Mohamadzadeh, M.; Reske, K.; Knop, J., Increased level of intracellular MHC class II molecules in murine Langerhans cells following in vivo and in vitro administration of contact allergens. *J Invest Dermatol* **1992,** *99* (5), 545-9.

17. Martinez Gomez, J. M.; Periasamy, P.; Dutertre, C. A.; Irving, A. T.; Ng, J. H.; Crameri, G.; Baker, M. L.; Ginhoux, F.; Wang, L. F.; Alonso, S., Phenotypic and functional characterization of the major lymphocyte populations in the fruit-eating bat Pteropus alecto. *Sci Rep* **2016,** *6*, 37796.
18. Marconato, L.; Gelain, M. E.; Comazzi, S., The dog as a possible animal model for human non-Hodgkin lymphoma: a review. *Hematol Oncol* **2013,** *31* (1), 1-9.
19. Forberg, H.; Hauge, A. G.; Valheim, M.; Garcon, F.; Nunez, A.; Gerner, W.; Mair, K. H.; Graham, S. P.; Brookes, S. M.; Storset, A. K., Early responses of natural killer cells in pigs experimentally infected with 2009 pandemic H1N1 influenza A virus. *PLoS One* **2014,** *9* (6), e100619.
20. O'Regan, A. W.; Hayden, J. M.; Body, S.; Liaw, L.; Mulligan, N.; Goetschkes, M.; Berman, J. S., Abnormal pulmonary granuloma formation in osteopontin-deficient mice. *Am J Respir Crit Care Med* **2001,** *164* (12), 2243-7.
21. Mitra, T.; Gerner, W.; Kidane, F. A.; Wernsdorf, P.; Hess, M.; Saalmuller, A.; Liebhart, D., Vaccination against histomonosis limits pronounced changes of B cells and T-cell subsets in turkeys and chickens. *Vaccine* **2017,** *35* (33), 4184-4196.
22. Wang, L.; Shen, D.; Wu, H.; Ma, Y., Resistance of hypervirulent Klebsiella pneumoniae to both intracellular and extracellular killing of neutrophils. *PLoS One* **2017,** *12* (3), e0173638.

Gene name	Primer sequences
**SNRPD3 (Es)**	TGGGAAGCTCATTGAGGCAG
	TGCCACGGATGTATACCTGC
**β actin (Es)**	GTCTTTCCCTCCATCGTGGG
	TCGATGGGGTACTTCAGGGT
**RPL4 (Es)**	ACCAAGGAGGCTGTTTTGCT
	CTCATTTTGCCCTTGCCAGC
**NOS2 (Es)**	GTTTGACGAGAGGACCCAGG
	TCTCCTTTGTCACCGCTTCC
**S100A12 (Es)**	AAAGATCAGCCCGCCATTGA
	ATGTTAACGTGGGCGGTCTT
**CYBB (Es)**	TAAGATAGCGGTTGACGGGC
	AATGGATGCGAAGGGTGTGA
**IL-10 (Es)**	AACACGTGAACTCCCTTGGG
	GCTCCACTGCCTTGCTCTTA
**IL-8 (Es)**	TTGGCAGCTTTCGTGCTTTC
	TGGGGTGAAAAGGTGCAGAA
**IL-1α (Es)**	GACACACTGCATGAGGACCA
	AACTCAGCCGCCTCTTCTTC
**IL-1β (Es)**	AGCAGCAACGAGAATGACCT
	GGAGACCTGCAGCTGGATG
**NLRP3 (Es)**	TGCTGCCATCAACCGTAGAG
	CAACAAGCCCATCCACTCCT
**CD88 (Es)**	CGACCGCTTTCTACTGGTGT
	GGACGGTATGATGAGCAGCA
**CD68 (Es)**	CAACAAAACCAAGGCCCAGG
	ATAGACAGTGCTCTGCGGTG
**CCR2 (Es)**	CACGTGGGTTATTTTGGCGG
	GGTGACCGTCCTGGCTTTTA
**MARCO (Es)**	TGGGACTGGAAACATCTGGC
	CATCGTGGTTGCAGTTGTGG
**CD14 (Es)**	ACGAACTGATGCTCGAGGAC
	CACGTTCCGGATACTGAGGG
**CD163 (Es)**	GGAGAGTGGGGAACTGTGTG
	AAATTCGTCCAGAGCCTGCA
**CD36 (Es)**	GCCCAGAGGTGCCATTTTTG
	GGATAGAGATGGGGTGCAGC
**CD206 (Es)**	TGACACGCTTTTGGGGATCA
	CGTAGATCTTCCACCGGCTC
**CD172α (Es)**	TGAGCTTCACCTGCAAGTCC
	GGTTGTAGGAAGCGCTGTCT

**CD169 (Es)**	GGACCCCAATCTGGTGGAAG
	ATTGTAGGAGCCCGAGTCCT
**SERPIN B2 (Es)**	CGGACCAAATGGCCAAAGTG
	AAGTGGCCTTCTGGATCTGC
**KLF4 (Es)**	CCCGTCCTTCTCCACGTTC
	GGAGTCGCTTCATGTGGGAG
**CFP (Es)**	AAGTGGAGACTGGGACCCTT
	GAGTCCGGGTTCCTTTGGAG
**TGFB1 (Es)**	GCTACTAGTGCTGACGCCTT
	CTTGGATAGGATCTGGCCGC
**IL-1RN (Es)**	TGCAAGCCTTCAGGATCTGG
	GCACCACACTGAGCTTCTCT
**CX3CR1 (Es)**	CCTCCTGAACTTGGCCTTGT
	TGCACACCGCGTTGTTAAAG
**SLC12A1 (Es)**	AGTACTATCGCAACACCGGC
	AACCCTGTCAGCTGAACCTG
**CD16 (Es)**	GCTCAGGAACGACAGTGTGA
	CGATGACGTAGCTGGAGGTC
**TNFα (Es)**	GCACTGAAAGCATGATCCGG
	CCACGAGGAGGAAGGAGAAG
**GAPDH (Hsa)**	AGGGCTGCTTTTAACTCTGGT
	CCCCACTTGATTTTGGAGGGA
**NOS2 (Hsa)**	ATGGGAGAAGGGGATGAGCT
	GAATGTGCTGTTTGCCTCGG
**IL-8 (Hsa)**	CTCCAAACCTTTCCACCCCA
	TTCCTTGGGGTCCAGACAGA
**TNFα (Hsa)**	CTCTCTGCCATCAAGAGCCC
	CACCCTTCTCCAGCTGGAAG
**GAPDH (Mmu)**	GGTCGGTGTGAACGGATTT
	CTGGAACATGTAGACCATGTAGTT
**NOS2 (Mmu)**	CTATGGCCGCTTTGATGTGC
	TTGGGATGCTCCATGGTCAC
**TNFα (Mmu)**	CACAGAAAGCATGATCCGCG
	GAGGCTGAGACATAGGCACC

Supplementary Table 3: Real-time PCR primers for detecting indicated target genes in *E. spelaea* (Es)*, H. sapiens* (Hsa) *and M. musculus* (Mmu)

Target protein	Antibody clone	Conjugate	Source
**CCR2**	SN707	Unconjugated	Novus Biologicals
**CD11c**	BU15	PE/Cy7	Biolegend
**CD11c**	3.9	BV605	Biolegend
**CD11c**	B-Ly6	BV650	BD Biosciences
**CD15**	C3D-1	PerCP-Cy5.	SCBT
**CD15**	W6D3	PerCP	Biolegend
**CD16**	KD1	FITC	Biorad
**CD18**	6.7	BV421	BD Biosciences
**CD19**	6D5	APC/Cy7	Biolegend
**CD26**	M-A261	BV510	BD Biosciences
**CD26**	JM11-42	Unconjugated	ThermoFisher Scientific
**CD29**	TS2/16	SB600	ThermoFisher Scientific
**CD31**	TLD-3A12	BV510	BD Biosciences
**CD33**	WM53	PE/CF594	BD Biosciences
**CD33**	P67.6	PercP/Cy5.5	BD Biosciences
**CD36**	FA6-152	FITC	Stemcell Technologies
**CD45**	F10-89-4	Biotin	Novus Biologicals
**CD45**	35-Z6	AF405	SCBT
**CD64**	10.1	AF700	BD Biosciences
**CD80**	2D10	BV421	Biolegend
**CD88**	P12/1	FITC	AbD Serotec
**CD88**	S5/1	PE/Cy7	Biolegend
**CD89**	A59	APC	Biolegend
**CD92**	VIM15	BUV395	BD Biosciences
**CD115**	AFS98	AF488	eBioscience/ThermoFisher Scientific
**CD115**	9-4D2-1E4	PerCP/Cy5.5	Biolegend
**CD117**	104D2	PercP/Cy5.5	BD Biosciences
**CD123**	6H6	BV650	Biolegend
**CD123**	7G3	BV650	BD Biosciences
**CD141**	AD5-14H12	PE/Vio770	Miltenyi
**CD163**	GHI/61	BV605	Biolegend
**CD163**	EDHu-1	AF647	Novus Biologicals

**CD169**	7-239	PE	BD Biosciences
**CD169**	HSn 7D2	AF700	Novus Biologicals
**CD172α**	CC149	RPE-Cy5	Biorad
**CD337**	P30-15	BV711	Biolegend
**S100A9**	MAC387	AF594	SCBT

Table S4: Details of antibodies which did not show clear cross-reactivity via flow cytometry analysis upon staining *E. spelaea* spleen and bone marrow tissue.
